# Metabolomic characterisation and flavour profiles of prawn, scallop, squid, barramundi, Salmon, snapper, and tuna

**DOI:** 10.1016/j.fochx.2025.102284

**Published:** 2025-02-14

**Authors:** Jiaqiang Luo, Damian Frank, Amanda L. Peterson, Brunda Nijagal, Jayashree Arcot

**Affiliations:** a Food and Health group, School of Chemical Engineering, Faculty of Engineering, UNSW Sydney, Kensington, NSW 2052, Australia; b All G, Waterloo, NSW 2017, Australia; c Metabolomics Australia, The University of Melbourne, Parkville, VIC, 3010, Australia

**Keywords:** Seafood, Flavour, Odourant, Tastant, Element

## Abstract

In this study volatile and non-volatile flavour metabolites were systematically measured in prawn, scallop, squid, barramundi, salmon, snapper, and tuna using headspace solid-phase microextraction gas chromatography–mass spectrometry for volatile compounds, liquid chromatography-mass spectrometry for non-volatile taste metabolites, and inductively coupled plasma mass spectrometry for elemental analysis. The data indicated that a core group of flavour compounds were commonly present across seafood species, contributing to fundamental seafood flavour. Orthogonal partial least squares-discriminant analysis identified unique odourants, tastants, and elements that differentiate individual species. These findings provide valuable insights for understanding differences in the flavour between key seafood species and in formulating more realistic plant-based seafood flavours.

## Highlights

• Seafood species can be differentiated by odourant, tastant and elemental profiles.
• Cooking significantly changes the volatile profiles of all investigated seafood species.
• Salmon has the most distinct odour characteristics.
• Prawn, scallop and squid have higher concentrations of taste compounds compared to finfish species.
• Prawn has the highest bromine and bromophenol content.

## 1 Introduction

Seafood is consumed globally in high volume and is highly valued for its nutritional content, which includes high-quality protein, polyunsaturated fatty acids (PUFAs), vitamins, and minerals and its unique texture and flavour. The Food and Agriculture Organization (FAO, 2022), estimated global seafood production at 178 million tonnes, with a total value exceeding USD 406 billion in 2020. The demand for seafood is expected to increase, exceeding the capacity of natural systems to sustainably meet demand. Global fish production, is projected to reach 202 million tonnes by 2032 (FAO, 2022). Notably, the widespread popularity of seafood is attributed not only to its nutritional benefits but also to its diverse and appealing flavour characteristics, which vary significantly across different aquatic creatures, each defined by its unique composition of flavour compounds.

A core group of odour-active volatile compounds are likely to play a crucial role in defining seafood flavour. The top odour active volatiles in cooked seafood from published olfactometry studies were identified as (*Z*)-4-heptenal, 2-acetyl-1-pyrroline, 2,3-butanedione, (*Z*)-1,5-octadien-3-one, hexanal and 2-ethylpyrazine (Luo et al., 2024). Furthermore, certain characteristic compounds can contribute unique attributes to seafood flavour. For example, timethylamine, widely associated with seafood odour, is generated from the degradation of trimethylamine oxide through enzymatic and bacterial activities. While higher concentrations of trimethylamine produce an unpleasant, rotten fishy smell, lower concentrations contribute a pleasant crustacean-like aroma (Cadwallader et al., 1995). Additionally, Luo et al. (2024) outlined influential odorants specific to various seafood categories. In crustaceans, sotolone, indole and a range of volatile acids, —e.g., 3-methylbutanoic acid, acetic acid, and decanoic acid —form the key odour-active compound profile. For molluscs, the prevalence of volatile compounds with roast nutty-flavour character, like 2-ethylpyrazine, 2-acetyl-2-thiazoline and 2-acetylpyrazine make them distinct compared to other seafood types. Finfish also show a varied odourant profile; oily fish is marked by earthy compounds like 1-octen-3-one, heptanal and 2-methylnaphthalene, whereas white fish are characterised by typical “fish-like” odorants such as (*E*)-2-nonenal, 1-hexanol, nonanal, and octanal.

Geosmin and 2-methylisoborneol are metabolites originating from aquatic cyanobacteria and actinobacteria, as well as fungi, and are responsible for imparting musty and earthy odour notes to seafood. Furthermore, 2-bromophenol, 4-bromophenol, 2,4-dibromophenol, and 2,4,6-tribromophenol, are often prevalent in wild seafood, particularly prawns and other crustaceans, and are noted for their contribution to authentic marine odours and their low odour thresholds, significantly enhancing the flavour of fresh wild seafood (Chung et al., 2003). However, these compounds are often missing in aquacultured seafood because they originate from algae accumulated by wild aquatic animals through their diets, a process not usually replicated in farmed seafood environments (Chung et al., 2003).

Seafood is generally cooked before consumption. During cooking, non-volatile flavour precursors are transformed by thermal reactions to yield a plethora of new flavour compounds. The oxidation of polyunsaturated fatty acids (PUFAs), for example, leads to a range of odour-active volatile compounds including aldehydes, ketones, and alcohols (Frank et al., 2015). Moreover, the thermal interactions between dicarbonyl compounds, including intermediates from sugar and lipid breakdown reactions, and amino acids through Strecker and Maillard reactions, generate a wide variety of odour-active volatiles. These include compounds like methional, 3-methylbutanal, 2,3-pentanedione, 2,3-butanedione, and various alkyl-pyrazines (Xu et al., 2013).

Seafood flavour is also determined by its taste quality, usually associated with savoury/umami sensations. This is mainly attributed to the presence of free glutamic acid and 5′-nucleotides such as inosine monophosphate (IMP), adenosine monophosphate (AMP) and guanosine monophosphate (GMP) in seafood (Sarower et al., 2012). Beyond umami, other free amino acids e.g., glycine, alanine, arginine may impart sour, bitter, or sweet tastes to the overall flavour spectrum of seafood (Shallenberger, 1993). Betaine (*N*,*N*,*N*-trimethyl glycine), a key osmolyte that protects cells from osmotic stress, may also contribute to the sweet and umami taste profile (Sarower et al., 2012). Moreover, sodium and potassium, minerals abundantly found in seafood, are linked to salty taste (Rotzoll et al., 2006). Furthermore, the synergistic effect of Na^+^ in enhancing umami taste have also been documented (Lioe et al., 2005).

Recent research has focused on identifying flavour compounds in commonly consumed seafood such as prawn (Duppeti et al., 2022), crab (Champion et al., 2022), and salmon (Duan et al., 2021). This body of work includes review articles that summarise key odour-active (Jones et al., 2022) or taste-active (Xin et al., 2023) compounds across multiple species, as well as our recent study that examined both odour- and taste-active compounds (Luo et al., 2024). However, due to the variability in analytical methods applied to analyse flavour compounds and the nature of the samples, particularly the differences arising from various cooking methods, a direct comparison of seafood species based on current published evidence is challenging. To address this gap, we surveyed commonly consumed seafood species in Australia, including barramundi (Asian seabass), salmon, snapper (seabream), and tuna, along with prawn, scallop, and squid. Following a standardised cooking procedure, seafood samples were then profiled for volatile compounds using headspace solid phase microextraction gas chromatography–mass spectrometry (HS-SPME-GC–MS), non-volatile taste metabolites using hydrophilic interaction liquid chromatography-mass spectrometry (HILIC–MS), and elements using inductively coupled plasma- mass spectrometry (ICP–MS) and ICP–optical emission spectroscopy (OES). To focus on compounds that significantly impact seafood flavour, we screened semi-quantified compounds from GC–MS and LC–MS analyses, selecting only those with a documented active contribution to flavour from literature. Odour-active compounds were chosen based on our previous research, which identified odourants that shape the characteristic aromas of crustaceans, molluscs, and finfish (Luo et al., 2024). From this work, we built a list of seafood odourants, including volatiles with the 20 highest relative odour activity values identified for each seafood type. For seafood tastants, our selection was guided by the studies of taste compounds in prawn (Bai et al., 2022; Duppeti et al., 2022; Zhang et al., 2024), scallop (Yin et al., 2022), salmon (Methven et al., 2007) and tuna (Zhang et al., 2019). Also considering the potential synergetic effects on taste perception, taste compounds with taste active values >0.5 were selected. Through statistical analyses, including orthogonal partial least-squares discriminant analysis (OPLS-DA) modelling, key flavour molecules defining different seafood species were identified.

## 2 Materials and methods

### 2.1 Chemical

4-Methyl-1-pentanol (99 %), 2-bromophenol (98 %), 2,6-dibromophenol (99 %), C7-C30 mixed alkane standard, methanol (99.9 %), ammonium carbonate (99.9 %), acetonitrile (ACN, hypergrade for LC–MS LiChrosolv®), ^13^C_6_-sorbitol (99 %), ^13^C_5_,^15^N_1_-Valine(99 %), ^13^C_10_^15^N_5_-AMP (adenosine monophosphate, 99 %), and ^13^C_9_^15^N_2_-UMP (uridine monophosphate, 99 %) were purchased from Merck (Castile Hill, NSW, Australia). Formic acid (Optima™ LC–MS grade) was purchased from Fisher Chemical™ (North Ryde, NSW, Australia). Purified water was obtained from a Milli-Q system (Millipore Australia, Bayswater, Victoria, Australia).

### 2.2 Seafood samples and sample preparation

Seafood samples were generally purchased fresh (not frozen) from the Sydney Fish Market (SFM) on different occasions over a five-month period (January–May 2023). Some scallop samples were purchased frozen. SFM strictly enforces truth-in-labelling, with the provenance and standardised name of seafood clearly displayed, therefore samples could be accurately identified (**Supplementary Table S1**). Seafood samples included: barramundi (Asian seabass, *Lates calcarifer*, *n* = 6); prawn (giant tiger prawn, *Penaeus monodon*, *n* = 3; tiger prawn, *Penaeus esculentus*, *n* = 2; king prawn, *Melicertus latisulcatus*, *n* = 1; salmon (Tasmanian Atlantic salmon, *Salmo salar*, n = 6); scallop (Hokkaido scallop, *Patinopectin yessoensis,* n = 2; Atlantic sea scallop, *Placopectin megallanicus*, n = 1; Ballots saucer scallop, *Amusium balloti*, n = 2; Southern scallop, *Pecten fumatus*, n = 2; snapper (silver seabream, *Pagrus auratus*, *n* = 4); squid (Loligo squid, *Loligo Formosa*, n = 1; Southern ocean calamari, *Sepioteuthis australis*, n = 3; Gould's squid, *Nototodarus gouldi*, n = 1), New Zealand arrow squid, *Nototodarus sloanii*, n = 1) and tuna (yellowfin tuna, *Thunnus albacares*, *n* = 7). Fish samples were purchased as fillets with skin on, where possible. Squid was immediately gutted, and the mantel was sliced into smaller sections. On the day of purchase a subsample (approximately 40 g) of the flesh of each species was ground into a fine paste with liquid nitrogen and a hand stick blender (Kitchen Aid, Benton Harbor, MI). Samples were transferred into labelled amber glass vials, sealed and frozen (−80 °C) until further chemical analysis. On the day of purchase, raw ground samples were transferred to headspace sampling vials for immediate volatile analysis. For cooked samples, seafood was grilled at 220 °C on a Roband Grill Station (Cromer, Australia) until an internal temperature of 70 °C was reached. The internal temperature was measured using a thermocouple placed in the geometric centre of samples. Fish fillets were salted and oiled (canola oil spray) on the skin side and cooked skin side down and then flipped. Prawns were cooked with heads and shells on, which were removed after grilling. Both raw and cooked samples were ground to mince using a food homogeniser. Samples for HS-GC–MS analysis were prepared fresh and temperately stored at 4 °C before analysis. For non-volatile profiling and elemental analysis, samples were frozen at −80 °C and subsequently thawed at 4 °C overnight prior to analysis.

### 2.3 HS-SPME-GC–MS analysis of volatiles in seafood

Three grams of seafood sample together with 10 μL of 4-methyl-1-pentanol internal standard (10 μg/mL) in 100 μL Milli-Q water in a 200 μL glass insert were added into a 20 mL headspace GC vial. Prior to the headspace extraction, the sample vial was equilibrated at 40 °C for 5 min with agitation using an incubation port attached to an AOC-6000 autosampler (Shimadzu, Kyoto, Japan). The extraction was conducted by exposing a 1.10 mm 120 μm DVB/carbon WR/PDMS SPME arrow fibre to the headspace at 40 °C for 40 min.

Seafood volatiles were analysed using a Shimadzu GC–MS-QP-2020 NX system. The SPME arrow was desorbed at 240 °C for 5 min in splitless mode. Chromatographic separation was performed by using a Zebron-Wax capillary column (Phenomenex, CA; 30 m × 0.25 mm ID × 0.25 μm) carried by purified helium (99.999 % purity, Coregas, New South Wales, Australia) in constant pressure mode at 100 kPa. GC oven was started at 35 °C and held for 2 min. The temperature was then increased to 250 °C at 6 °C/min, followed by a final holding time of 22 min. Mass spectrometry was operated in positive electron ionisation (EI) mode at 70 eV, scanning ions ranged from *m/z* 35 to 220. Ion source and interface temperatures were 200 °C and 240 °C, respectively.

A C7-C30 mixed alkane standard was run to obtain retention indices (RIs) of volatiles. Tentative compound identification was based on the comparisons of EI mass spectra and RIs to the records in National Institute of Standards and Technology (NIST), United States of America, library (version 11). Peak integration was based on the response of target ions. Semi-quantification was facilitated by the internal standard and semi-quantitative results were expressed in μg 4-methyl-1-pentanol/g fresh sample. Details of compound identification and quantification were summarised in **Supplementary file 1**.

### 2.4 Analysis of bromophenols in prawn samples

No clear bromophenol signal was detected in other species during our pilot experiments, therefore, the analysis was conducted solely on prawn samples. Prawn samples were divided into head and flesh portions and were processed and cooked using the same procedure described in section 2.2 prior to analysis.

The GC–MS conditions and semi-quantification approach remained unchanged for the analysis of bromophenols, except that the range of ion scanning was extended to *m/z* 35 to 350. In addition to matching mass spectra with library records, the identification of bromophenols was further facilitated by using authentic standards of 2-bromophenol and 2,6-dibromophenol.

### 2.5 Determination of non-volatile compounds in seafood by LC–MS

Tissue optimisation was conducted to determine the optimal biomass of tissue required to provide the best metabolite coverage in each of the seafood samples. The optimal mass (60 mg) of tissue were extracted by homogenisation in a Precellys® 24 Tissue homogeniser coupled to a Cryolys® cooling system (Bertin Technologies) for liquid chromatography–mass spectrometry (LC–MS) analysis by adding 600 μL of ice-cold 3:1 methanol: Milli-Q water containing a mixture of isotopically labelled internal standards from different chemical classes- (^13^C_6_-sorbitol, ^13^C_5_,^15^N_1_-Valine, ^13^C_10_^15^N_5_-AMP (adenosine monophosphate), and ^13^C_9_^15^N_2_-UMP (uridine monophosphate). Samples were filtered using a Captiva EMR-lipid plate (Agilent Technologies) prior to LC–MS analysis.

Metabolite separation was performed on Vanquish Horizon UHPLC system (Thermo Scientific, Waltham, MA) coupled to an Orbitrap ID-X Tribrid mass spectrometer (Thermo Scientific) for metabolite detection by Metabolomics Australia (MA, University of Melbourne). The chromatography conditions were modified from Lu et al. (2023). In brief, separation was performed on Merck SeQuant ZIC-pHILIC column (150 mm × 4.6 mm, 5 μm particle size) maintained at 25 °C, using a binary gradient consisting of solvent A: 20 mM ammonium carbonate (pH 9.0; Sigma–Aldrich) and solvent B: 100 % ACN. The gradient run was as follows: time (t) = 0.0 min, 80 % B; *t* = 0.5 min, 80 % B; *t* = 15.5 min, 50 % B; *t* = 17.5 min, 30 % B; *t* = 18.5 min, 5 %; *t* = 21.0 min, 5 % B; *t* = 23–33 min, 80 % at a solvent flow rate of 300 μL/min.

Metabolite detection was performed on a Orbitrap ID-X Tribrid Mass Spectrometer (Thermo Scientific) coupled to a heated electrospray ionisation (H-ESI) source with the following conditions: sheath gas flow 40 arbitrary units (Arb), auxiliary gas flow 10 Arb, sweep gas flow 1 Arb, ion transfer tube temperature 275 °C, and vaporizer temperature 320 °C. The RF lens value was 35 %. Data was acquired in negative polarity with spray voltages of 3500 V.

Data analysis was performed on TraceFinder 4.1 General Quan Software (Thermo Scientific) and El-Maven v0.12.1 (https://www.elucidata.io/el-maven). Level 1 metabolite identification, according to the Metabolite Standard Initiative (Summer et al., 2007), was based on matching accurate mass and retention time to the 550 authentic standards in the Metabolomics Australia (MA) in-house library. Details of identified compounds were summarised in **Supplementary file 2**.

### 2.6 Elemental analysis by ICP–MS and ICP–OES

Elemental analysis was conducted by ICP Laboratory, Solid State & Elemental Analysis Unit, UNSW, Sydney. Open acid digestion was performed before analysis of Ca, K, Mg, Na and P. In brief, 0.1 g of seafood sample was digested by 200 μL of concentrated HNO_3_ for 12 h at room temperature followed by heating in a boiling water bath for 2 h. After that, the digestate was made up to 10 mL with MQ water ready for ICP–OES analysis. For the analysis of Br and I, 0.2 g sample was digested with 10 mL of 2.5 % TMAH at a 90 °C water bath for 3 h with a tight cap. After diluting to 50 mL with MQ water and centrifugation, the supernatant was analysed by ICP–MS.

### 2.7 Statistics

After auto-scaling, fold change analysis and *t*-test of raw and cooked volatiles were conducted to investigate the influence of heat treatment on the seafood volatile profile using MetaboAnalyst version 6.0 (https://www.metaboanalyst.ca). The result was visualised in a volcano plot with a fold change (FC) threshold of 2 and *p*-value threshold at 0.001 false discovery rate (FDR). One-way analysis of variance (ANOVA) was conducted to compare differences in mean values of compounds amongst seafood groups. Principal component analysis (PCA) was performed to investigate sample discrimination based on selected odourants and tastants screened as descript in the introduction. Relationships between volatiles measured in cooked seafood with non-volatile flavour precursors in raw seafood (protein, omega-3 PUFAs, metabolites and elements) were analysed by Pearson correlation analysis. Statistical tests as above were performed and visualised using XLSTAT software version: 2023.2.0 (1411) (Lumivero, Denver, CO). Orthogonal partial least squares-discriminant analysis (OPLS-DA) was conducted by grouping individual species as one group and all other species as the other, repeating the analysis for datasets of odourants, tastants, and elements using MetaboAnalyst. Concentrations of odourants, tastants, and elements were auto-scaled prior to modelling. Models were constructed separately for each dataset and were validated by 5-fold cross validation method with performance measured by accuracy, R^2^ and Q^2^. Flavour compounds and elements with variable importance in projection (VIP) scores >1 in OPLS-DA models were defined as key flavour compounds and were summarised and visualised in sunburst charts created with Python (v5.20.0) using the sunburst chart function in the Plotly Graphing Libraries.

## 3 Results and discussion

### 3.1 Volatile composition of raw and cooked seafood

**Fig. 1:**
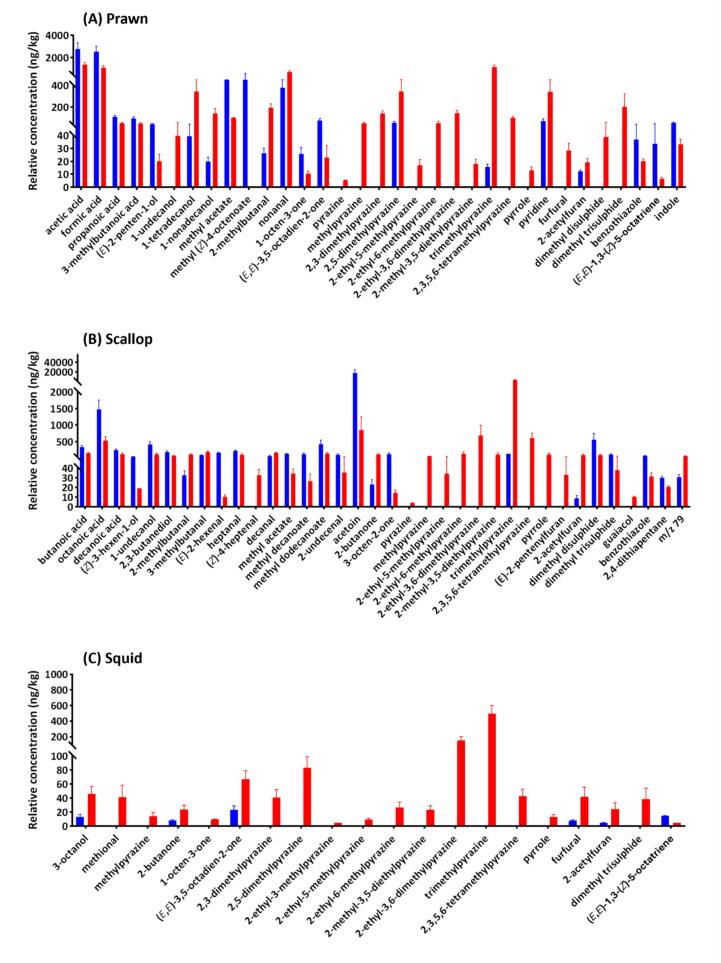

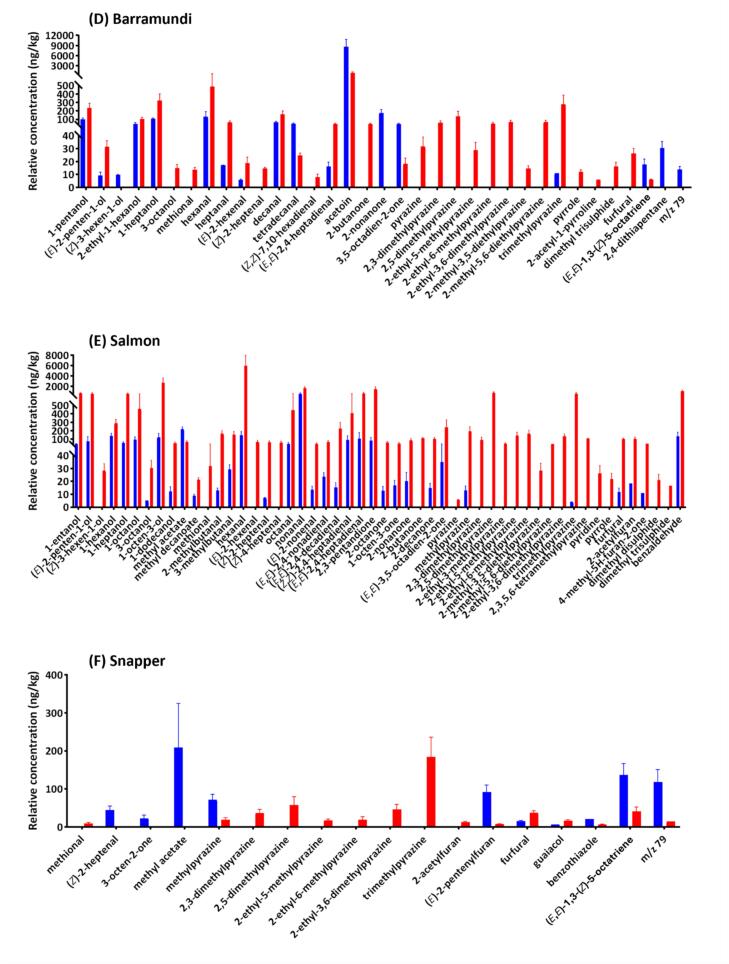

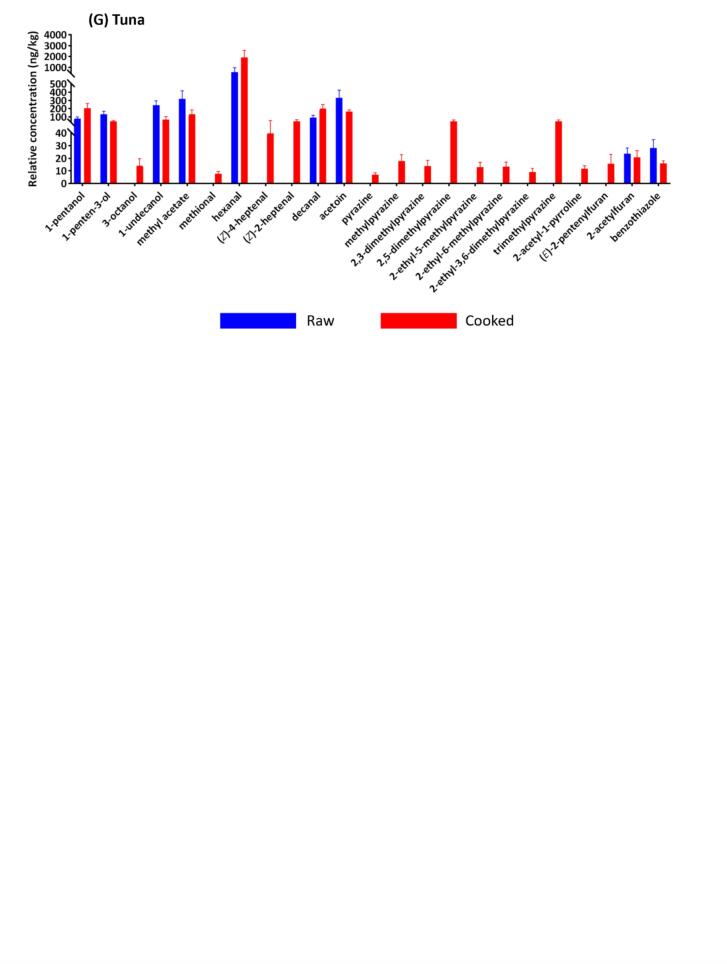
Variation in volatile composition between raw and cooked seafood samples. Histograms show compounds with significant differences (*p* < 0.05) between replicate raw (blue) and cooked (red) samples for (**A**) prawn, (**B**) scallop, (**C**) squid, (**D**) barramundi, (**E**) salmon, (**F**) snapper and (**G**) tuna. Standard deviations are represented by error bars. Volatile compounds were ordered according to class and them retention index. Volatile compounds were ordered according to class and then by retention index. (For interpretation of the references to colour in this figure legend, the reader is referred to the web version of this article.)

One hundred and thirteen volatiles were tentatively identified and semi-quantified from HS-SPME-GC–MS analysis across our cooked and raw seafood samples (**Supplementary File 1**). Following a fold change analysis between all raw and cooked samples (**Supplementary Fig. S2A**) and comparison within individual species (Fig. 1A-G), a range of acids, alcohols and esters were found more pronounced in raw seafood in general. Most of these compounds are associated with the microbial and enzymatic activities that occur within the marine animals or generated during storage (Jones et al., 2022). Scallop was particularly rich in alcohols (**Supplementary Fig. S2B**), which could be attributed to the specific biochemical composition of scallop as well as the difference in diet and aquatic environment compared to other species, leading to stronger microbial and enzymatic activities that promote the production of volatile alcohols. In comparison, cooked seafood samples were characterised by a higher presence of aldehydes, pyrazines, pyrroles, pyridines, furans and sulphides, which are typically generated due to heat treatment (Nieva-Echevarría et al., 2017). Notably, a significantly higher pyrazine level was found in scallop (**Supplementary Fig. S2C**). While seafood generally lacks carbohydrates, scallops have the highest carbohydrate content amongst the investigated seafood species, serving as an important substrate for pyrazine production (**Supplementary Table S3**). This may account for the high abundance of pyrazines observed in cooked scallop. This overall trend of changes in volatile composition after cooking aligns with findings from our previous research (Luo et al., 2024) and has been reported in numerous other studies; therefore, it is not further elaborated upon here. Instead, the following section focuses on some species-specific changes in odourant composition.

Significant (*p* < 0.05) decreases in many volatile alcohol, acid, ketone and sulphide compounds after cooking were widely found in prawn (Fig. 1A), scallop (Fig. 1B), squid (Fig. 1C), barramundi (Fig. 1D), snapper (Fig. 1F) and tuna (Fig. 1G). The loss of these compounds was attributed to the evaporation during cooking. Conversely, for most of these compounds in salmon (Fig. 1E), their concentrations increased after cooking. Salmon contains a relatively high level of omega-3 PUFA (**Supplementary Table S3**), which breaks down into smaller volatile molecules when heated.

A significant (*p* < 0.05) increase in indole content was only observed in prawn (Fig. 1A). Indole plays an important role in the flavour profile of prawn and is produced through the microbial metabolism of tryptophan (Li & Young, 2013). Although high concentrations of indole can indicate spoilage, when present at lower concentrations it may contribute to a desirable prawn flavour profile (Whitfield, 1999). Our findings indicate a decrease in indole content after cooking. Interestingly, Mall and Schieberle (2016) detected a high concentration of indole in blanched prawn meat, but not in raw or fried prawn, suggesting that boiling may promote indole production. These observations imply that indole content in prawn is susceptible to the cooking method, which could be related to the specific time, temperature, and medium in direct contact with the prawn meat.

An analysis targeting four bromophenols in prawns was conducted, with the results summarised in Table 1. Although 2,6-dibromophenol was present only at trace levels in our samples and below the detection limit, the results indicated that 4-bromophenol was the most prevalent form of bromophenol in prawns, followed by 2,4-dibromophenol and 2-bromophenol. Additionally, in agreement with Whitfield et al. (1997), our results demonstrated that, in both raw and cooked samples, prawn heads had higher concentrations of bromophenols compared to the flesh. Moreover, a significant reduction in bromophenols due to heat treatment was observed, a finding that has not been reported previously. Given that bromophenols have relatively high boiling points and/or high logP values (Table 1), their loss during cooking is likely attributed mainly to drip loss.

**Table 1:** Semi-quantitative concentrations of detected bromophenols in the heads and flesh of raw and cooked prawn samples.

			head		flesh	
	boiling point (°C)	LogP	raw	cooked	raw	cooked
2-bromophenol	194.5	2.4	33.1 a	20.3 ab	0 b	2.0 b
4-bromophenol	238.0	2.6	555.2 a	51.6 b	133.7 ab	6.5 b
2,4-dibromophenol	238.5	3.2	271.3 a	9.5 b	12.0 b	0.2 b
						

**Notes:** Values are expressed as ng /kg fresh sample. For each bromophenol, different letters indicate statistically significant differences (*p* < 0.05) in mean values compared by one-way ANOVA followed by Fisher's post hoc test. 2,6-dibromophenol and 2,4,6-tribromophenol were not detected in any samples. Boiling points and logP values indicating compound solubility are from https://pubchem.ncbi.nlm.nih.gov/.

Dimethyl disulphide and dimethyl trisulphide levels in scallop decreased after cooking, which differs from barramundi (Fig. 1D) and salmon (Fig. 1E), where significant increases in sulphide compounds were observed. Scallop is relatively low in protein and sulphur amino acids (**Supplementary Table S3**), and it is likely that these two sulphides were lost by volatilisation during cooking.

Additionally, an unidentified volatile compound, characterised by a prominent *m/z* 79 ion (RI: 1425) was tenetatively identified as 2,7-octadien-1-ol based on its MS match with the NIST library, was consistently detected across various seafood types, particularly in cooked scallops. This may suggest that scallop was particularly rich in the precursor(s), such as certain PUFAs.

Salmon had the largest number of significant changes in volatile compounds after cooking compared to the other seafood species (Fig. 1E). Of note, except for methyl acetate, all other compounds increased significantly. Apparently, this is associated with the fact that salmon has the highest unsaturated fat and omega-3 PUFA content. In comparison, fewer compounds changed significantly after cooking in squid (Fig. 1C), snapper (Fig. 1F), and tuna (Fig. 1G). This could be associated with their relatively low concentration of non-volatile odourant precursors (**Supplementary Table S3**). Additionally, as the cooking procedure was standardised based on internal temperature, the fat and protein compositions, as well as the size and thickness of the portions, could affect the cooking time. Therefore, significant changes in volatiles might not be observable in samples with short cooking times.

Correlation analysis of the relationship between non-volatile flavour precursors - protein, omega-3 PUFAs, metabolites, and elements indicated significant (*p* < 0.05) positive correlations with volatile degradation products (**Supplementary File 3**). This well-known relationship aligns with our observations, as discussed in the section above. Additionally, other significant (*p* < 0.05) correlations were identified, such as the positive correlation of sodium and bromine with various free amino acids, negative correlations between potassium and some free amino acids and negative correlations between taurine and a range of omega-3 PUFAs degraded products. These findings may suggest potential modulatory interactions between volatiles, non-volatiles, and elements. However, no causal relationships have been established.

### 3.2 Discrimination of seafood samples based on odourants

The observed variations in the volatile composition of different raw seafood samples could offer valuable insights into understanding the differences in odour perception when seafood is consumed raw, such as in sashimi. However, seafood is typically cooked before consumption. In addition, only odour-active volatile compounds are likely to play a role in the flavour profile. Therefore, a subset of volatiles (*n* = 45, **Supplementary File 1**), which were previously identified as key seafood odourants (Luo et al., 2024), were selected for the statistical analysis of cooked seafood samples.

**Fig. 2:**
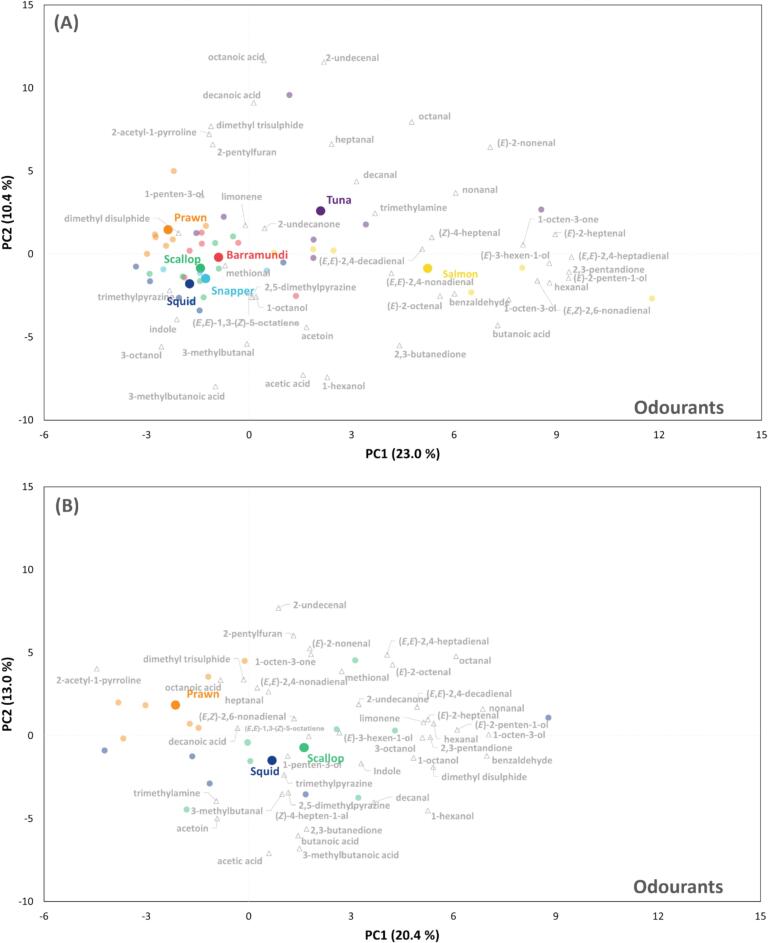

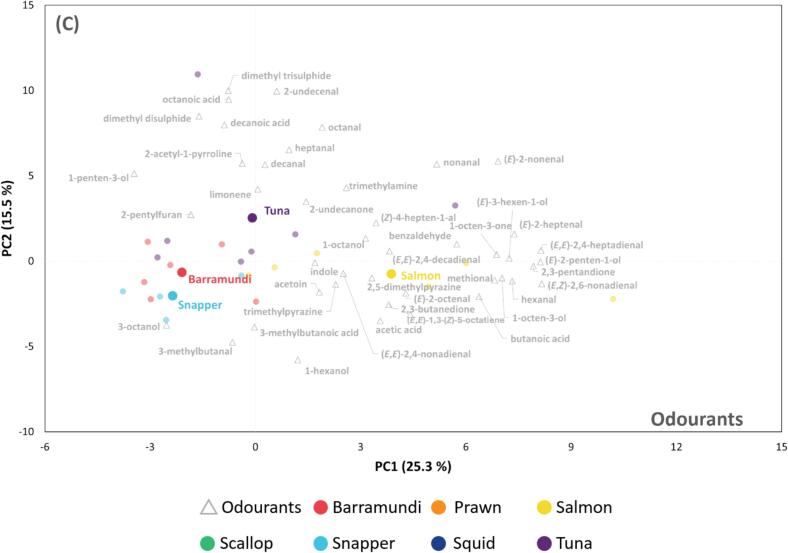
Multivariate analyses of odourant profiles of investigated seafood species. PCA biplots illustrate the separation and clustering of (**A**) prawn, scallop, squid, barramundi, salmon, snapper and tuna samples, (**B**) prawn, scallop and squid samples and (**C**) four fish samples. Prawn, scallop, squid, barramundi, salmon, snapper and tuna replicates are labelled in orange, green, dark blue, red, yellow, light blue and purple, respectively. Odourant names are labelled in grey. (For interpretation of the references to colour in this figure legend, the reader is referred to the web version of this article.)

As shown in PCA biplot based on all seven investigated seafood species (Fig. 2A), 33.4 % of total variance was explained by the first two components with the majority separation achieved in PC1 (23.0 %). Two oily fish, salmon and tuna were found in the positive side of PC1 strongly driven by typical compounds derived from the PUFA breakdown including (*E*,*E*)-2,4-heptadienal, 2,3-pentandione, (*E*)-2-penten-1-ol, (*E*)-2-heptenal, hexanal, (*E*)-3-hexen-1-ol, (*E*,*Z*)-2,6-nonadienal, 1-octen-3-one, 1-octen-3-ol and butanoic acid. Of note, methional, (*E*,*E*)-1,3-(*Z*)-5-octatriene, 1-octanol, 2,5-dimethylpyrazine, limonene and 2-undecanone sit close to the origin, indicating these compounds were commonly found across our seafood species and not significantly different in concentration. Therefore, it is reasonable to speculate that these compounds form the basis of generic, cooked “seafood-like” odour.

Although a distinct separation of prawn, scallop, squid, barramundi, and snapper is not observed in Fig. 2A, a separate PCA of the three non-fish species reveals characteristic odourants (Fig. 2B). Prawn was differentiated from squid and scallop in the space defined by negative PC1 and positive PC2, primarily due to its relatively high level of 2-acetyl-1-pyrroline, a volatile typically found at high concentrations in prawns (Mall & Schieberle, 2016; Mall & Schieberle, 2017). Furthermore, 2-acetyl-1-pyrroline has also been identified as a key odourant in squid, noted for its high concentration in squid broth (Carrascon et al., 2014). This explained the positioning of most squid samples on the negative side of PC1. Squid was separated from the prawn cluster due to higher concentrations of acids (acetic, 3-methylbutanoic, and butanoic acids) and ketones (acetoin and 2,3-butanedione), which drove squid samples towards the negative side of PC2. Scallop samples were associated with a higher concentration of alcohols such as 1-octen-3-ol, (*E*)-2-penten-1-ol, 1-hexanol and 1-octanol as well as aldehydes such as nonanal, benzaldehyde, hexanal and (*E*)-2-heptenal. This result agrees with previous studies, which reported that alcohols and aldehydes were the most abundant classes of volatile compounds in scallop (Chen et al., 2022).

A more detailed odourant characterisation of four fish species was conducted by repeating PCA without the inclusion of prawn, scallop, and squid samples (Fig. 2C). The clearest differentiation was observed for the salmon samples. The other samples, particularly the two white fish species barramundi and snapper, were not well-separated. This result implied that mainly volatiles derived from PUFA were the key determinants differentiating the odour profiles of fish species.

### 3.3 Discrimination of seafood samples based on non-volatile tastants

**Fig. 3:**
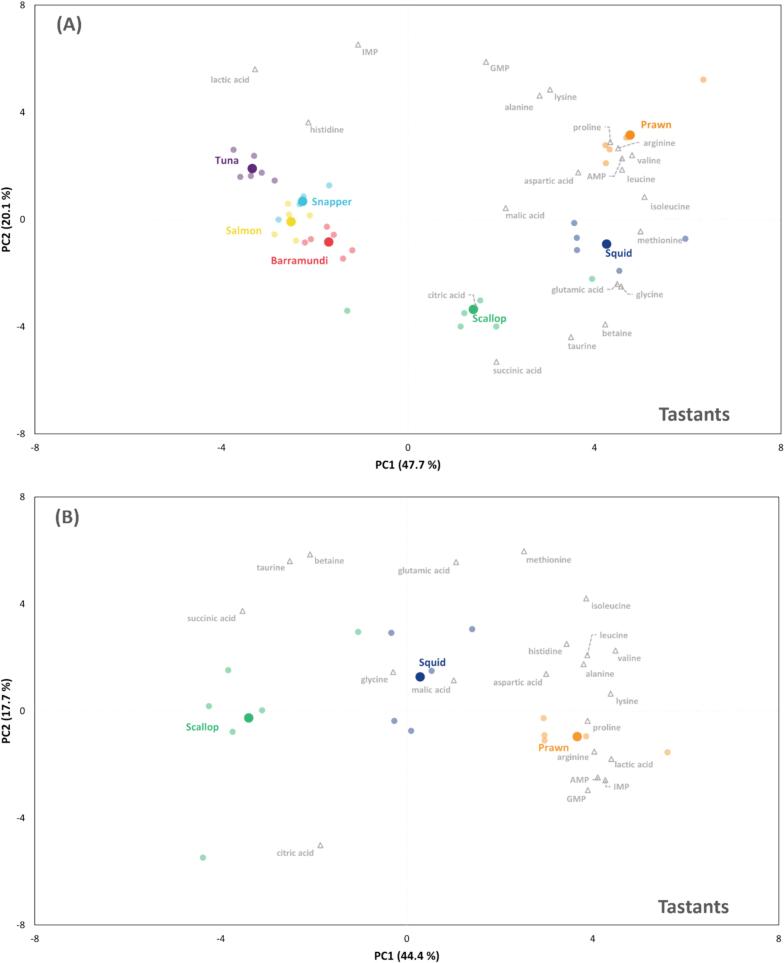

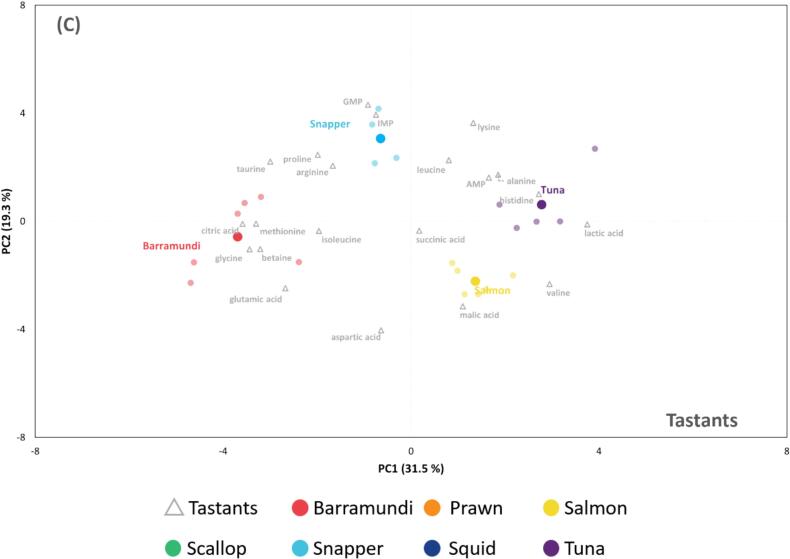
Multivariate analyses of selected flavour active tastants of investigated seafood species. PCA biplots illustrate the separation and clustering of (**A**) prawn, scallop, squid, barramundi, salmon, snapper and tuna samples, (**B**) prawn, scallop and squid samples and (**C**) four fish samples. Prawn, scallop, squid, barramundi, salmon, snapper and tuna are labelled in orange, green, dark blue, red, yellow, light blue and purple, respectively. Tastant names are labelled in grey. (For interpretation of the references to colour in this figure legend, the reader is referred to the web version of this article.)

A total of 173 non-volatile compounds were identified following LC–MS analysis. Referring to the work on seafood taste-active compounds, 21 non-volatile compounds were selected for further multivariate analysis (**Supplementary file 2**). Compared to the PCA of odourants, the analysis based on tastants achieved better separation and clearer clustering of individual groups, with the first two PCs explaining a higher total variance (67.8 %). The separation of four fish and three non-fish samples in PC1 (Fig. 3A) was significantly attributed to a range of free amino acids, particularly isoleucine, methionine, valine, leucine, glycine, and glutamic acid. Furthermore, while prawn, squid, and scallop were further differentiated in PC2, the four fish species tended to cluster together, indicating a more similar tastant profile across the fish species.

Analysis targeted at prawn, scallop and squid (Fig. 3B) revealed that prawn samples differed from the other two species owing to higher levels of 5′-nulceotides including IMP, GMP and AMP, free amino acids including arginine and proline, as well as lactic acid. While squid samples mainly distributed around the origin, scallop samples were found in the negative side of PC1, mainly associated with higher levels of organic acids such as succinic and citric acids, taurine and betaine.

Following the removal of non-fish samples, well-separated clusters of fish species were observed (Fig. 3C). Barramundi samples were clustered on the negative side of PC1, which was influenced by citric acid, glycine, methionine and betaine. Snapper samples were typically characterised by higher GMP and IMP contents, forming a distinct cluster in the positive side of PC2. Two oily fish distributed on the positive sides of PC1, with tuna characterised by high lactic acid, histidine, alanine and AMP contents, and salmon which was associated with high levels of valine and malic acid. Furthermore, succinic acid was located next to the origin, suggesting that it is a compound commonly found in fish and may play a generalist role in fish flavour.

The exact reasons for the varying composition of these non-volatile compounds across species remain unclear. However, they have been associated with differences in environmental adaptation, dietary pattern, metabolism and muscle composition (Gao et al., 2021). For instance, barramundi, the only euryhaline fish amongst the species studied, has the highest betaine content. This is likely linked to barramundi's need to transition between freshwater and marine environments and to tolerate a wide range of salinities. A rich reservoir of osmolytes, including betaine, are necessary to maintain cellular homeostasis under fluctuating osmotic pressures. Furthermore, the demanding swimming lifestyle of tuna explains the high concentrations of lactic acid and histidine found in this species. Histidine, along with carnosine and anserine, are required to ensure muscle buffering capacity, mitigating the harmful effects of lactic acid accumulation during burst swimming (Abe et al., 1986).

Xin et al. (2023) reported that trimethylamine oxide was the most important metabolite distinguishing aquatic from livestock and poultry products. However, trimethylamine oxide was not detected in our analysis owing to its amine-like structure that can only produce a positive charge. The HILIC analysis was performed in negative mode, with the conditions promoting the generation of negative charge and hence could not ionise trimethylamine oxide. While lacking a few compounds that are exclusively detectable using positive ion mode in acidic conditions, it has been proven that the negative ion mode generally has better sensitivity over a broader range of compounds (Liigand et al., 2017). Besides, trimethylamine oxide possesses a high taste threshold of 1 g/100 g (Rotzoll et al., 2006), and there is no evidence to suggest its presence at levels exceeding this threshold in seafood (**supplementary Table S4**). Therefore, it is unlikely that excluding trimethylamine oxide from our flavour analysis could significantly affect the understanding of the overall flavour profile of our samples.

### 3.4 Comparison of elemental composition

The analysis of taste-related elements revealed significant differences in sodium, magnesium, phosphorus, potassium calcium and bromine contents (Table 2). Briefly, prawn had the highest sodium and magnesium concentrations, and snapper was particularly rich in phosphorus, potassium and calcium. Furthermore, intermediate concentrations of minerals studied were observed in scallop, barramundi and salmon. Mineral elements are linked to the salty taste and can synergistically contribute to umami taste (Lioe et al., 2005).

**Table 2:** Concentrations of selected elements measured by inductively coupled plasma spectrometry in seafood samples.

	Prawn (*n* = 6)	Scallop (*n* = 6)	Squid (*n* = 5)	Barramundi (*n* = 5)	Salmon (*n* = 7)	Snapper (*n* = 4)	Tuna (*n* = 6)
Sodium (mg/g)	3.2 a	1.5 b	2.8 a	0.6 cd	0.3 d	0.9 c	0.4 cd
Magnesium (mg/g)	0.5 ab	0.4 b	0.4 c	0.3 d	0.3 d	0.3 cd	0.5 a
Phosphorus (mg/g)	3.3 b	2.8 c	2.6 d	1.7 g	2.4 e	3.5 a	1.9 f
Potassium (mg/g)	3.4 b	3.6 b	1.6 c	3.5 b	3.8 b	4.5 a	4.5 a
Calcium (mg/g)	0.8 b	0.3 c	0.1 c	0.2 c	0.1 c	1.2 a	0.2 c
Bromine (μg/g)	4.8 a	1.3 b	0.2 b	0.6 b	0.1 b	0.5 b	0.3 b

**Notes:** For each element, different letters indicate statistically significant differences (*p* < 0.05) in mean values compared by one-way ANOVA followed by Fisher's post hoc test.

Our focus on bromine was due to its known prevalence in marine creatures, primarily in the form of bromophenols. Therefore, it is reasonable to infer that the overall bromine content closely correlates with the presence of bromophenols and the pleasant marine-like flavour notes they impart. Consistent with our HS-SPME-GC–MS analysis, which detected bromophenols only in prawn samples, the highest bromine content was found in prawns. Likewise, other studies have reported higher bromophenol contents in prawn samples compared to finfish (Zhang et al., 2012). In addition, while not statistically significant, scallop had a higher bromine content compared with squid and all fish species. Marine creatures do not synthesise bromine-containing compounds endogenously. Instead, the presence of bromines in marine creatures is largely attributed to environmental and dietary sources, such as algae (Chung et al., 2003). Given that prawns and scallops often inhabit algae-rich coastal waters and estuaries, this accounts for their observed higher bromine as well as bromophenols content.

### 3.5 Determination of key compounds for flavour characterisation of investigated seafood by OPLS-DA

**Fig. 4:**
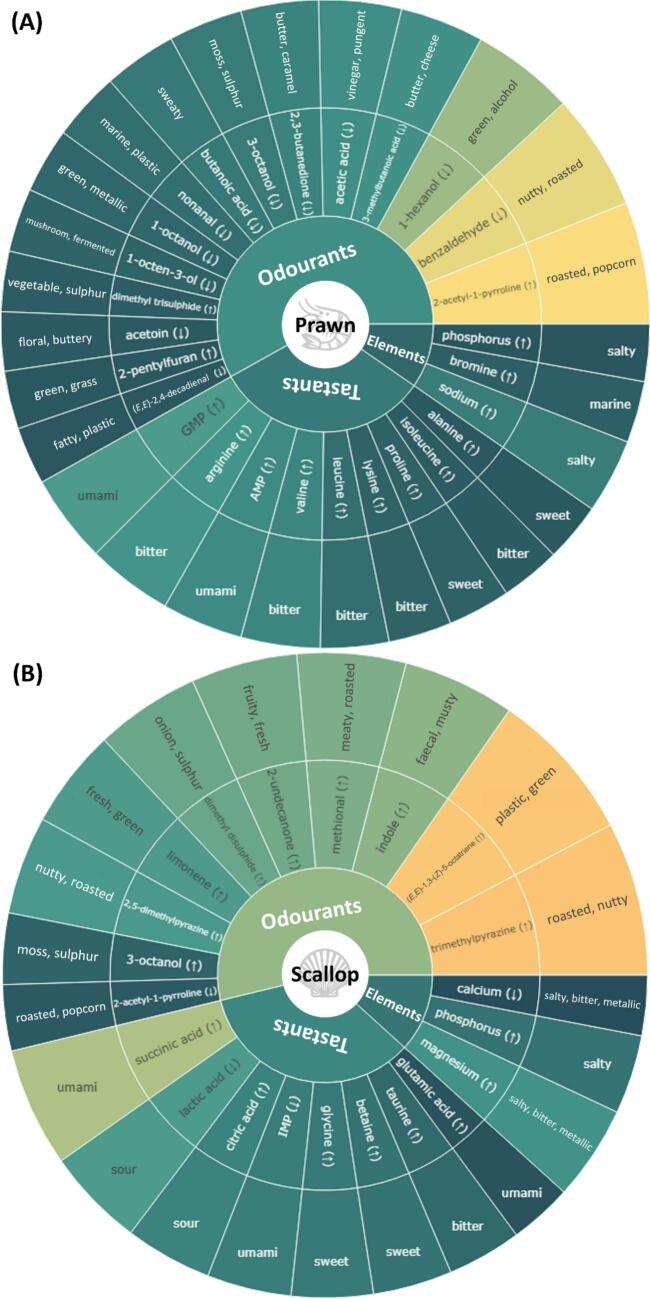

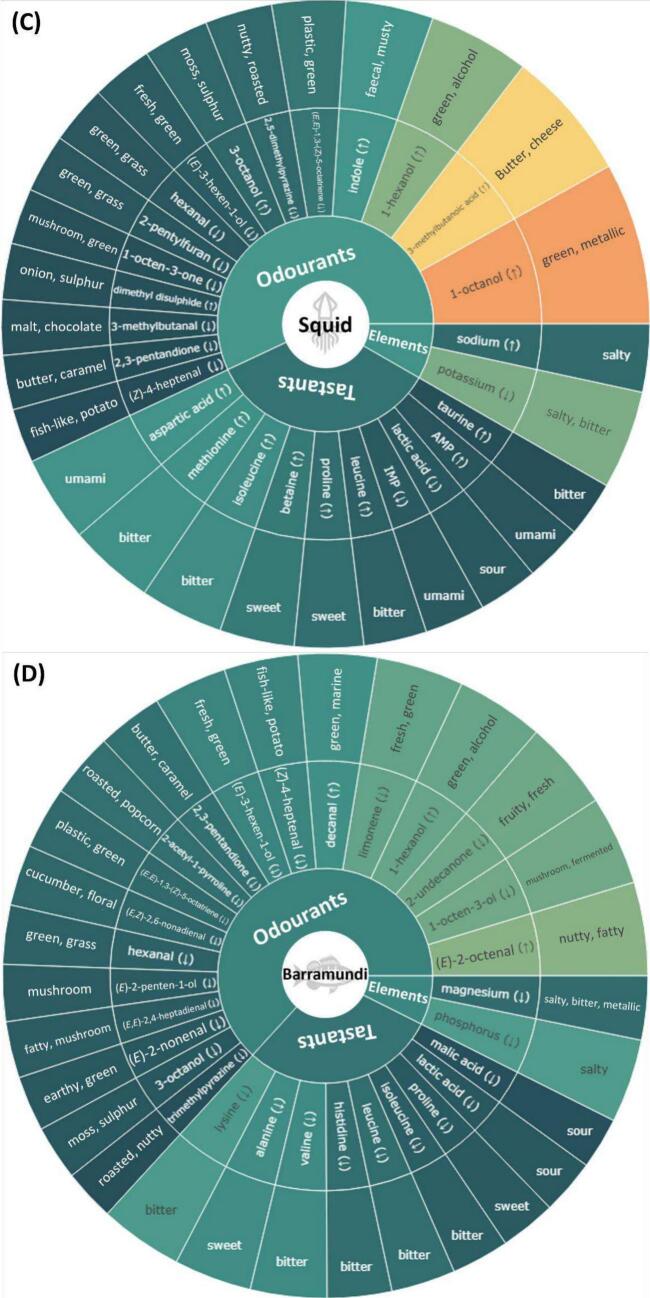

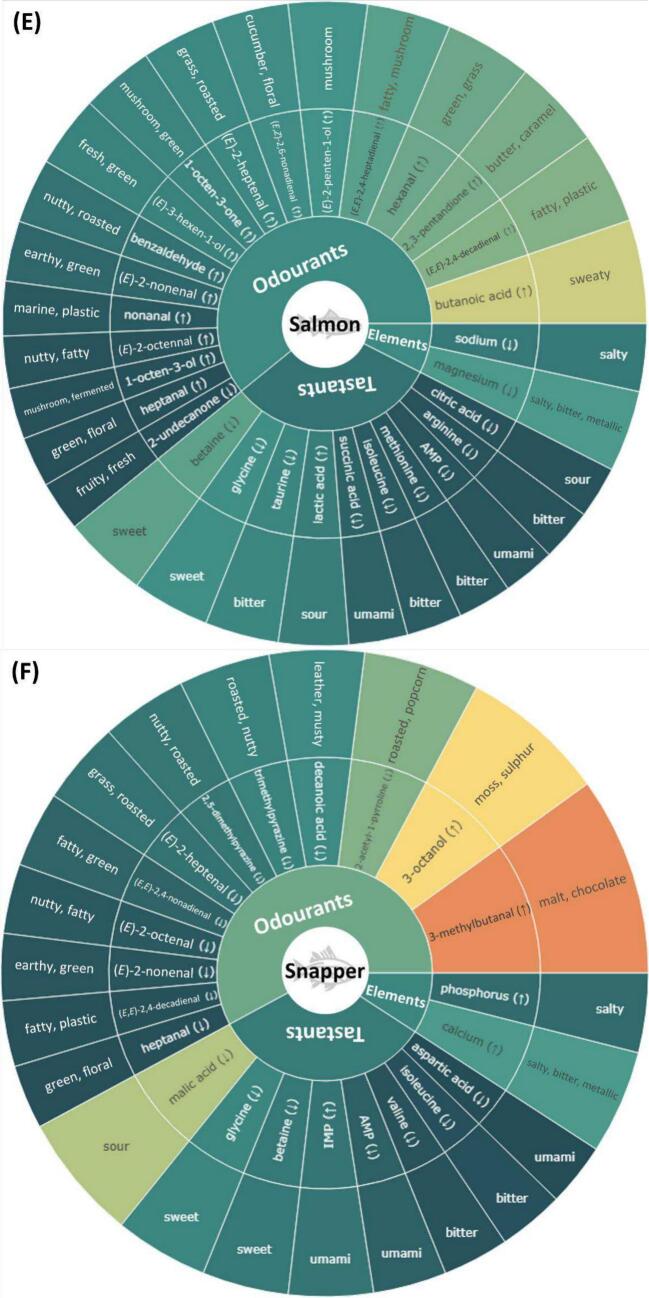

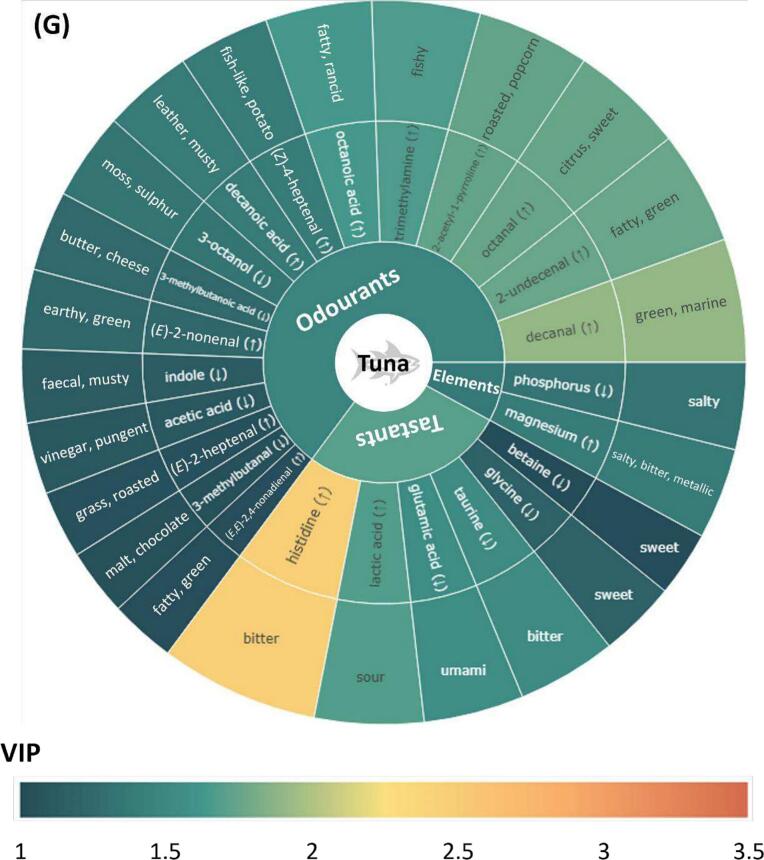
Flavour wheels illustrating key odourants, tastants and inorganic ions in seafood species. The wheels display the most distinctive (variable importance projection (VIP) scores >1) odourants, tastants, and inorganic ions for (**A**) prawn, (**B**) scallop, (**C**) squid, (**D**) barramundi, (**E**) salmon, (**F**) snapper and (**G**) tuna based on VIP scores from OPLS-DA models. VIP scores are labelled using a gradient from dark green to red, indicating a range from low to high values. Upper and lower arrows indicate whether the compound/element is particularly higher or lower in that species compared to others. (For interpretation of the references to colour in this figure legend, the reader is referred to the web version of this article.)

Previous studies have typically emphasised compounds that have high abundance or high odour-active values when evaluating their contribution to the flavour profile of a species (Duan et al., 2021; Duppeti et al., 2022). However, without a comparison across species, compounds that make the flavour of a species unique remain unclear. Additionally, the typical flavour of a seafood species is not only attributed to the presence or abundance of individual compounds but can also be due to the lack or deficiency of others. To address this, OPLS-DA was conducted by grouping individual species as one group and all other species as the other. As shown in **Supplementary Figs. S5–11**, most of the OPLS-DA models had good sample separation and clustering. Additionally, high Q^2^ values and significant permutation test scores (empirical *p*-value <0.05) were found in most models, indicating high accuracies and low possibilities of overfitting. According to the contribution to species characterisation, as reflected by VIP scores, key flavour compounds and elements (VIP > 1) for each species were ranked and presented in the form of flavour wheels (Fig. 4A-G). Notably, compounds and elements could have high VIP scores due to their particularly high or low content in a species compared to others, which was highlighted with “↑” and “↓” in the flavour wheels. To better visualise the flavour contribution, descriptors were included in outer rings of flavour wheels as well as the following discussion. For odourants, the two most frequently reported descriptors summarised by Jones et al. (2022) were selected. Descriptors of tastants and elements were based on the work of Duppeti et al. (2022), Bai et al. (2022) and Lawless et al. (2003).

In general, odourants occupied the largest proportion in the flavour wheel, followed by tastants and elements, indicating the dominant roles of odour-active compounds in characterising seafood flavour. Common flavour compounds and elements were found across the seafood species. For example, 3-octanol (*moss, sulphur*) is present in all flavour wheels except that of salmon; betaine (*sweet*), isoleucine (*bitter*), and lactic acid (*sour*) are present in the flavour wheels of at least five species, and phosphorus (*salty*) was identified as a key flavour element for prawn, scallop, barramundi, snapper, and tuna. Key odourants and tastants combinations unique to specific species are summarised in subsequent sections. Since our targeted elements, except bromine, are all associated with the salty taste and bromine appeared only in the flavour wheel of prawns, which supports the important role of bromophenols in prawn flavour as discussed, taste-contributing elements are not further elucidated below.

#### 3.5.1 Prawn

The odourant profile of prawn was characterised by a high abundance of 2-acetyl-1-pyrroline (*roasted, popcorn*), dimethyl trisulphide (*vegetable, sulphur*) and 2-ethylfuran (*green, grass*) while low abundance of acids, alcohols, aldehydes and ketones (Fig. 4A). 2-Acetyl-1-pyrroline was the most influential odourant in the prawn odour profile. This aligns with the research by Mall and Schieberle (2017) and Mall and Schieberle (2016), which identified 2-acetyl-1-pyrroline as the most significant odour-active compound in both blanched and fried prawn meat with the highest odour active value across all of their quantified volatile compounds. Dimethyl trisulphide and 2-ethylfuran were also identified as key odour compounds unique to and abundant in prawn. While they are well-recognised key odour-active compounds in crab (Champion et al., 2022), their contribution to prawn flavour has not been reported by olfactometry studies. Therefore, the role of dimethyl trisulphide and 2-pentylfuran in the flavour spectrum of prawns deserves further investigation.

Two 5’nucleotides (GMP and AMP,) and the sweet amino acids proline and alanine were found to be key tastants in the flavour profile of prawns, owing to their higher concentration compared to other species. Their presence at above taste threshold levels in prawns has previously been reported by Bai et al. (2022) and Zhang et al. (2024). Several bitter amino acids, including arginine, valine, leucine, lysine, and isoleucine, also distinguished prawns from other species. However, owing to their relative high taste thresholds, no studies have yet found that these amino acids are taste-active in prawns as well as in most of other seafood species. Therefore, the contribution of bitter amino acids to the flavour profile of seafood remains to be proven.

#### 3.5.2 Scallop

The flavour wheel of scallop consists of odourants with significantly higher concentrations in the species compared to others, including trimethylpyrazine (*roasted, nutty*), (*E*,*E*)-1,3-(*Z*)-5-octatriene (*plastic, green*), indole (*faecal, musty*), methional (*meaty, roasted*), 2-undecanone (*fruity, fresh*), dimethyl disulphide (*onion, sulphur*), limonene (*fresh, green*), 2,5-dimethylpyrazine (*nutty, roasted*) and 3-octanol (Fig. 4B). On the contrary, scallop had relatively low concentrations of 2-acetyl-1-pyrroline. Of note, methional was a prominent volatile in the flavour wheel of scallop. Apart from the direct provision of the “meaty” and “roasted” notes, Toda et al. (2018) demonstrated a positive allosteric modulation effect of methional on an umami taste receptor (T1R1/T1R3). Therefore, it is possible that the umami taste of scallop, contributed by umami compounds like free amino acids and 5′-nucleotides, is enhanced by methional. Besides, while there is a lack of GC–O studies on scallops with quantitative scores (e.g., flavour dilution factor and direct intensity), the role of (*E*,*E*)-1,3-(*Z*)-5-octatriene in shaping the flavour of turbot (*Psetta maxima*) has been reported (Sérot et al., 2001). Given its high VIP score in the flavour wheel, the odour impact of (*E*,*E*)-1,3-(*Z*)-5-octatriene on scallop is worthy of more detailed investigation.

Scallop was particularly rich in succinic and glutamic acids,imparting umami taste as well as citric acid (*sour*), glycine (*sweet*), betaine, and taurine (*bitter*). On the other hand, it was characterised by lower lactic acid (*sour*) and IMP (*umami*) contents.

#### 3.5.3 Squid

The key odour profile of squid consisted of alcohols including 1-octanol (*green, metallic*), 1-hexanol (*green, alcohol*) and 3-octanol, 3-methylbutanoic acid (*butter, cheese*), indole and dimethyl disulphide, which were found at higher concentrations compared to other species (Fig. 4C). On the contrary, particularly low levels of (*E*,*E*)-1,3-(*Z*)-5-octatriene, 2,5-dimethylpyrazine (nutty, roasted), (*E*)-3-hexen-1-ol (*fresh, green*), hexanal (*green, grass*), 2-pentylfuran (*green, grass*), 1-octen-3-one (*mushroom, green*), 2,3-pentadione (butter, caramel) and (*Z*)-4-heptenal (fish-like, potato) were observed. Squid had a key tastant profile with high levels of aspartic acid (*umami*), betaine, proline, AMP and four bitter amino acids (methionine, isoleucine, leucine and taurine) while low in IMP and lactic acid.

#### 3.5.4 Barramundi

While barramundi had the highest number of key odourants (*n* = 18), only (*E*)-2-octenal (*nutty, fatty*), decanal (*green, marine*), and 1-hexanol were highlighted due to their relatively high concentrations compared to other species (Fig. 4D). Similarly, tastants in the barramundi flavour wheels were chosen due to their lower concentrations This finding may account for the observation by Lawley et al. (2021) that barramundi frequently gave consumers an impression of a light flavoured fish.

#### 3.5.5 Salmon

A total of 17 key odourants were identified in salmon, with 16 showing a higher abundance compared to other species (Fig. 4E). In agreement, these compounds were also found odour-active in a previous study on cooked salmon (Milo & Grosch, 1996). On the contrary, salmon typically lacked tastants except for lactic acid. This suggested that the distinctive flavour of salmon was primarily due to the retronasal and orthonasal olfaction of its rich and diverse volatile compounds, rather than its taste properties. The higher overall fat content is likely to also affect the in-mouth volatile partitioning and release (Frank et al., 2015).

#### 3.5.6 Snapper

A range of PUFA-derived volatile compounds were identified as key odourants in snapper. However, except for 3-methylbutanal (*malt, chocolate*) and 3-octanol (*moss, sulphur*), all other compounds were found at lower concentrations in snapper compared to other species (Fig. 4F). In terms of tastants, snapper displayed a similar pattern to salmon, with only IMP being present in higher amounts, while the other seven tastants were lower.

#### 3.5.7 Tuna

Tuna had high concentrations of volatiles derived from heat treatment, including decanal (*green, marine*), 2-undecenal (*fatty, green*), octanal (*citrus, sweet*), 2-acetyl-1-pyrroline, octanoic acid (*fatty, rancid*), (*Z*)-4-heptenal (*fish-like, potato*), decanoic acid (*leather, musty*), (*E*)-2-nonenal (*earthy, green*), (*E*)-2-heptenal (*grass, roasted*), and (*E*,*E*)-2,4-nonadienal (*fatty, green*), which aligned with the finding of Zhang et al. (2019) in cooked tuna. Amongst these, 2-undecenal, octanal, and octanoic acid were unique key odourants of tuna (Fig. 4G). Additionally, trimethylamine (*fish-like, ammonia*) also appeared only in the tuna flavour wheel. Considering that our samples were purchased, processed, and analysed promptly, and no unpleasant fishy odour was detected during this process, trimethylamine in cooked tuna samples likely contributed a pleasant fish-like aroma (its odour quality at low concentrations) (Cadwallader et al., 1995). Furthermore, the key odourant profile for tuna was also characterised by low levels of 3-octanol, indole, acetic acid (*vinegar, pungent*) and 3-mehtylbutanal (*malt, chocolate*).

While having a relatively low concentration of most tastants, tuna had higher levels of histidine (*bitter*) and lactic acid compared with other species, which were associated with its unique swimming habits as discussed. The high levels of endogenous lactic acid in tuna, which was also reported by Zhang et al. (2019), may contribute to the subtle sour taste in tuna, especially for tuna sashimi.

## 4 Conclusion

For the first time, the same analytical profiling techniques were applied to elucidate the key odourants, tastants, and flavour-contributing elements across economically important and popular seafood species. It is widely recognised that each type of seafood possesses unique flavours, and this study identified some key molecular differences behind these distinct characteristics.

Starting with an investigation of the effect of cooking on volatile composition, we confirmed significant differences in the volatile profiles of raw and cooked seafood, unsurprisingly characterised by a general shift towards thermally induced lipid oxidation and Maillard reaction products. Focusing on cooked seafood, odourants including methional, (*E*,*E*)-1,3-(*Z*)-5-octatriene, 1-octanol, 2,5-dimethylpyrazine, limonene, and 2-undecanone were commonly found across species at relatively similar concentrations. Additionally, succinic acid (*umami*) was commonly found in four fish species. These compounds may play a fundamental role in defining the baseline seafood flavour.

Importantly, significant differences in flavour profiles between species were identified. Salmon exhibited the most distinct odourant profile due to its high abundance of odour precursors – overall lipid and PUFA content. Additionally, both GC–MS and elemental analysis confirmed the importance of bromophenols in prawn flavour. Furthermore, the loss of bromophenols in prawn head and the flesh during cooking was confirmed. Distinct tastant profiles were observed, with prawn, scallop, and squid having higher concentrations of taste compounds compared to fish species. Differences in the content of flavour-relevant elements was also observed, which could be directly associated with different saltiness levels. Key flavour compounds of the individual species were summarised in their corresponding flavour wheels.

These findings provide valuable insights into the flavourants driving the differentiation of various types of seafood. Beyond achieving a better understanding of typical seafood flavours from a chemical composition perspective, these insights could help overcome one of the major barriers to greater consumer acceptance of plant-based seafood— and their often-disappointing sensory quality (Luo et al., 2025). In general, plant-based seafood products are already highly flavoured and importantly, their flavour does not change significantly after heating. A better approach may lie in replicating natural thermally induced flavour pathways, whereby non-volatile flavour precursors are formulated to better mimic the profiles of seafood species, which can be transformed into volatiles upon heating. This approach may produce a more natural and acceptable solution. With the growing demand for more desirable and palatable plant-based seafood products, the information presented in this study may offer a practical starting point for formulating plant-based seafood flavours with more natural and convincing sensory attributes, closer to their authentic counterparts.

## CRediT authorship contribution statement

**Jiaqiang Luo:** Writing – review & editing, Writing – original draft, Visualisation, Methodology, Investigation, Formal analysis, Conceptualization. **Damian Frank:** Writing – review & editing, Writing – original draft, Supervision, Resources, Methodology, Funding acquisition, Conceptualisation. **Amanda L. Peterson:** Writing – review & editing, Methodology, Formal analysis. **Brunda Nijagal:** Writing – review & editing, Methodology, Formal analysis. **Jayashree Arcot:** Writing – review & editing, Supervision, Resources, Conceptualisation.

## Declaration of competing interest

The authors declare that they have no known competing financial interests or personal relationships that could have appeared to influence the work reported in this paper.

## Data Availability

LC–MS data can be accessed in the online repository: https://data.mendeley.com/datasets/yms4s58gbb/1. Other data are available from supplementary files.

## Appendix A Supplementary data

Multimedia Component 1 Supplementary tables and figures. Supplementary material 1

Multimedia Component 2 Supplementary file 1. Volatile composition. Supplementary material 2

Multimedia Component 3 Supplementary file 2. Non-volatile composition. Supplementary material 3

Multimedia Component 4 Supplementary file 3. Correlation analysis. Supplementary material 4
