# Supplementary material for: Metabolomic characterisation and flavour profiles of prawn, scallop, squid, barramundi, Salmon, snapper, and tuna
Source: Food Chem X. 2025 Feb 14;26:102284. doi: 10.1016/j.fochx.2025.102284 (PMC11905853; doi:10.1016/j.fochx.2025.102284)
Supplement: Multimedia Component 1 — Supplementary tables and figures. [file mmc1.docx]

**Supplementary Table S1.** Seafood sample information.

|  | **Common name** | **Species** | **Date of purchase** | **Purchased from** | **Status** | **Origin** |
| --- | --- | --- | --- | --- | --- | --- |
| Barramundi-A | barramundi | *Lates calcarifer* | 20-Dec-22 | local supermaket | fillet, skin on | Australia |
| Barramundi-B | barramundi | *Lates calcarifer* | 1-Feb-23 | SFM | fillet, skin off | Australia |
| Barramundi-C | barramundi | *Lates calcarifer* | 27-Apr-23 | SFM | fillet, skin on | Australia |
| Barramundi-D | barramundi | *Lates calcarifer* | 11-May-23 | SFM | fillet, skin on | Australia |
| Barramundi-E | barramundi | *Lates calcarifer* | 11-May-23 | SFM | fillet, skin on | Australia |
| Barramundi-F | barramundi | *Lates calcarifer* | 26-Jul-23 | SFM | fillet, skin on | Australia |
| Prawn-A | tiger prawn | *Penaeus esculentus* | 18-Jan-23 | local supermaket | meat, tail off | Australia |
| Prawn-B | tiger prawn | *Penaeus esculentus* | 20-Jan-23 | local supermaket | meat, tail off | Australia |
| Prawn-C | giant tiger prawn | *Penaeus monodon* | 1-Feb-23 | SFM | whole | Australia |
| Prawn-D | giant tiger prawn | *Penaeus monodon* | 2-Feb-23 | SFM | whole | Australia |
| Prawn-E | giant tiger prawn | *Penaeus monodon* | 27-Apr-23 | SFM | whole | Australia |
| Prawn-F | jumbo king prawn | *Melicertus plebejus* | 27-Apr-23 | SFM | whole | Australia |
| Salmon-A | Tasmanian salmon | *Salmo salar* | 12-Jan-23 | local supermaket | fillet, skin on | Australia |
| Salmon-B | Tasmanian salmon | *Salmo salar* | 12-Jan-23 | local supermaket | fillet, skin off | Australia |
| Salmon-C | Tasmanian salmon | *Salmo salar* | 1-Feb-23 | SFM | fillet, skin off | Australia |
| Salmon-D | Tasmanian salmon | *Salmo salar* | 27-Apr-23 | SFM | fillet, skin off | Australia |
| Salmon-E | Tasmanian salmon | *Salmo salar* | 11-May-23 | SFM | fillet, skin on | Australia |
| Salmon-F | Tasmanian salmon | *Salmo salar* | 26-Jul-23 | SFM | fillet, skin off | Australia |
| Scallop-A | Southern scallop | *Pecten fumatus* | 12-Jan-23 | local supermaket | roe off | Australia |
| Scallop-B | Ballots saucer scallop | *Amusium balloti* | 27-Apr-23 | SFM | roe off | Australia |
| Scallop-C | Hokkaido scallop | *Patinopectin yessoensis* | 11-May-23 | SFM | roe off | Japan |
| Scallop-D | Southern scallop | *Pecten fumatus* | 11-May-23 | SFM | roe on | Australia |
| Scallop-E | Ballots saucer scallop | *Amusium balloti* | 11-May-23 | SFM | roe off | Australia |
| Scallop-F | Hokkaido scallop | *Patinopectin yessoensis* | 26-May-23 | SFM | roe off | Japan |
| Scallop-G | Atlantic Sea Scallop | *Placopectin megallanicus* | 26-Jul-23 | SFM | roe off | Canada |
| Snapper-A | silver seabream | *Pagrus auratus* | 27-Apr-23 | SFM | fillet, skin on | Australia |
| Snapper-B | silver seabream | *Pagrus auratus* | 11-May-23 | SFM | fillet, skin on | Australia |
| Snapper-C | silver seabream | *Pagrus auratus* | 26-May-23 | SFM | fillet, skin off | Australia |
| Snapper-C | silver seabream | *Pagrus auratus* | 26-Jul-23 | SFM | fillet, skin on | Australia |
| Squid-A | Loligo squid | *Loligo formosa* | 27-Apr-23 | SFM | whole | Australia |
| Squid-B | Southern ocean calamari | *Sepioteuthis australis* | 27-Apr-23 | SFM | whole | Australia |
| Squid-C | Southern ocean calamari | *Sepioteuthis australis* | 11-May-23 | SFM | whole | Australia |
| Squid-D | Gould’s squid | *Nototodarus gouldi* | 11-May-23 | SFM | whole | Australia |
| Squid-E | Southern ocean calamari | *Sepioteuthis australis* | 26-May-23 | SFM | whole | Australia |
| Squid-F | arrow squid | *Nototodarus sloanii* | 26-Jul-23 | SFM | whole | New Zealand |
| Tuna-A | yellowfin tuna | *Thunnus albacares* | 1-Feb-23 | SFM | fillet, skin off | Australia |
| Tuna-B | yellowfin tuna | *Thunnus albacares* | 1-Feb-23 | SFM | fillet, skin off | Australia |
| Tuna-C | yellowfin tuna | *Thunnus albacares* | 27-Apr-23 | SFM | fillet, skin off | Australia |
| Tuna-D | yellowfin tuna | *Thunnus albacares* | 27-Apr-23 | SFM | fillet, skin off | Australia |
| Tuna-E | yellowfin tuna | *Thunnus albacares* | 11-May-23 | SFM | fillet, skin off | Australia |
| Tuna-F | yellowfin tuna | *Thunnus albacares* | 11-May-23 | SFM | fillet, skin off | Australia |
| Tuna-G | yellowfin tuna | *Thunnus albacares* | 1-Aug-23 | SFM | fillet, skin off | Australia |

**Abbreviation:** SFM, Sydney seafood market.


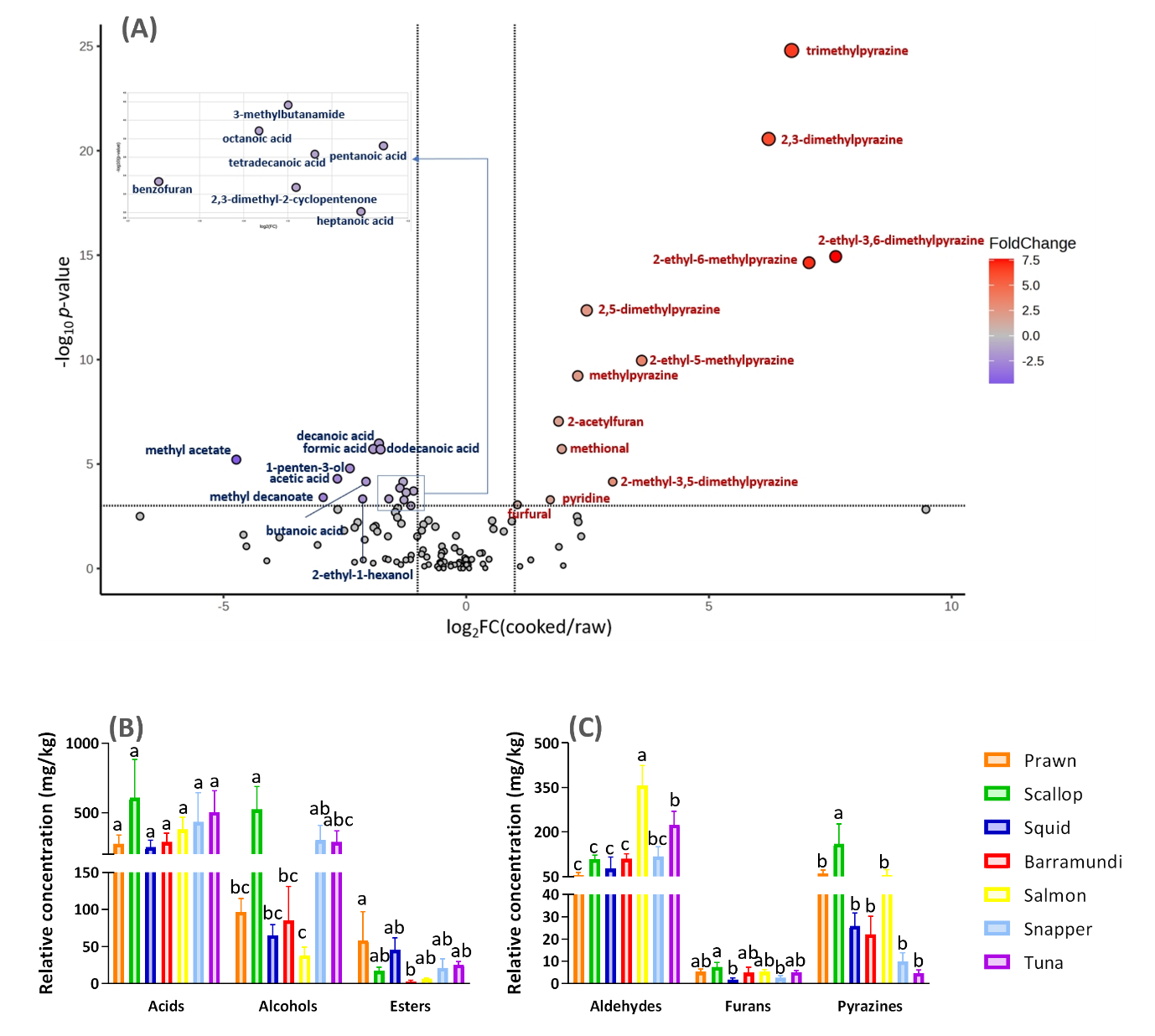


**Supplementary Figure S2.** Variation in volatile composition between raw and cooked seafood samples (**A**). Compounds labelled in blue and red are significantly different between the two groups. The colour gradient from blue to red correlates with the degree of fold change. Seafood species were significantly different (*p < 0.05*) in total alcohol and ester contents in raw (**B**) and in total aldehyde, furan and pyrazine contents after cooking (**C**). Prawn, scallop, squid, barramundi, salmon, snapper and tuna are labelled in orange, green, dark blue, red, yellow, light blue and purple, respectively.

**Supplementary Table S3.** Total omega-3 fatty acids and protein contents**.**

|  | Total omega-3 fatty acids  (mg/g fresh weight) | Carbohydrate  (%, fresh weight) | Protein  (%, fresh weight) |
| --- | --- | --- | --- |
| Barramundi | 5.4 | 0 | 19.4 |
| Prawn | 1.4 | 0 | 22.8 |
| Salmon | 28.1 | 0 | 21.9 |
| Scallop | 2.4 | 3.4 | 14.4 |
| Snapper | 2.7 | 0 | 19.7 |
| Squid | 3.3 | 0 | 12.5 |
| Tuna | 2.4 | 0 | 25.1 |

**Notes:** Total omega-3 fatty acid and carbohydrate contents were sourced from FSANZ database available at <https://afcd.foodstandards.gov.au/>. Shown contents were based on records (or mean values when multiple entries of the seafood type are available) of raw samples. Entry details are as follow: barramundi (F000386), prawn (F007422, F007424, F007431, F007433, F007451, F007454), salmon (F007827, F007849), scallop (F008166), snapper (F008359), Squid (F008828), tuna (F009303). Protein contents were obtained from pooled experimental samples analysed by elemental combustion analysis with a nitrogen conversion factor of 6.25.

**Supplementary Table S4.** Literature values of TMAO contents of investigated seafood types.

|  | Species | Method | Concentration  (g/100 g fresh weight) | Reference |
| --- | --- | --- | --- | --- |
| Prawn | mixed species from different depths | Spectrophotometer | 0.2-0.7 | (Kelly & Yancey, 1999) |
| Scallop | *Pecten maximus* | Flow injection analysis | 0.01 | (Ruiz-Capillas et al., 2001) |
| Squid | mixed species from different depths | Spectrophotometer | 0.8 | (Kelly & Yancey, 1999) |
| Barramundi | *Lates calcarifer* | HPLC | 0.1 | (Chung & Chan, 2009) |
| Salmon | *Salmo salar* | HPLC | 0.04 | (Chung & Chan, 2009) |
| Snapper | *Lutjanus malabaricus* | HPLC | 0.3 | (Chung & Chan, 2009) |
| Tuna | *Thunnus albacares* | NMR | 0.09 | (Jääskeläinen et al., 2019) |


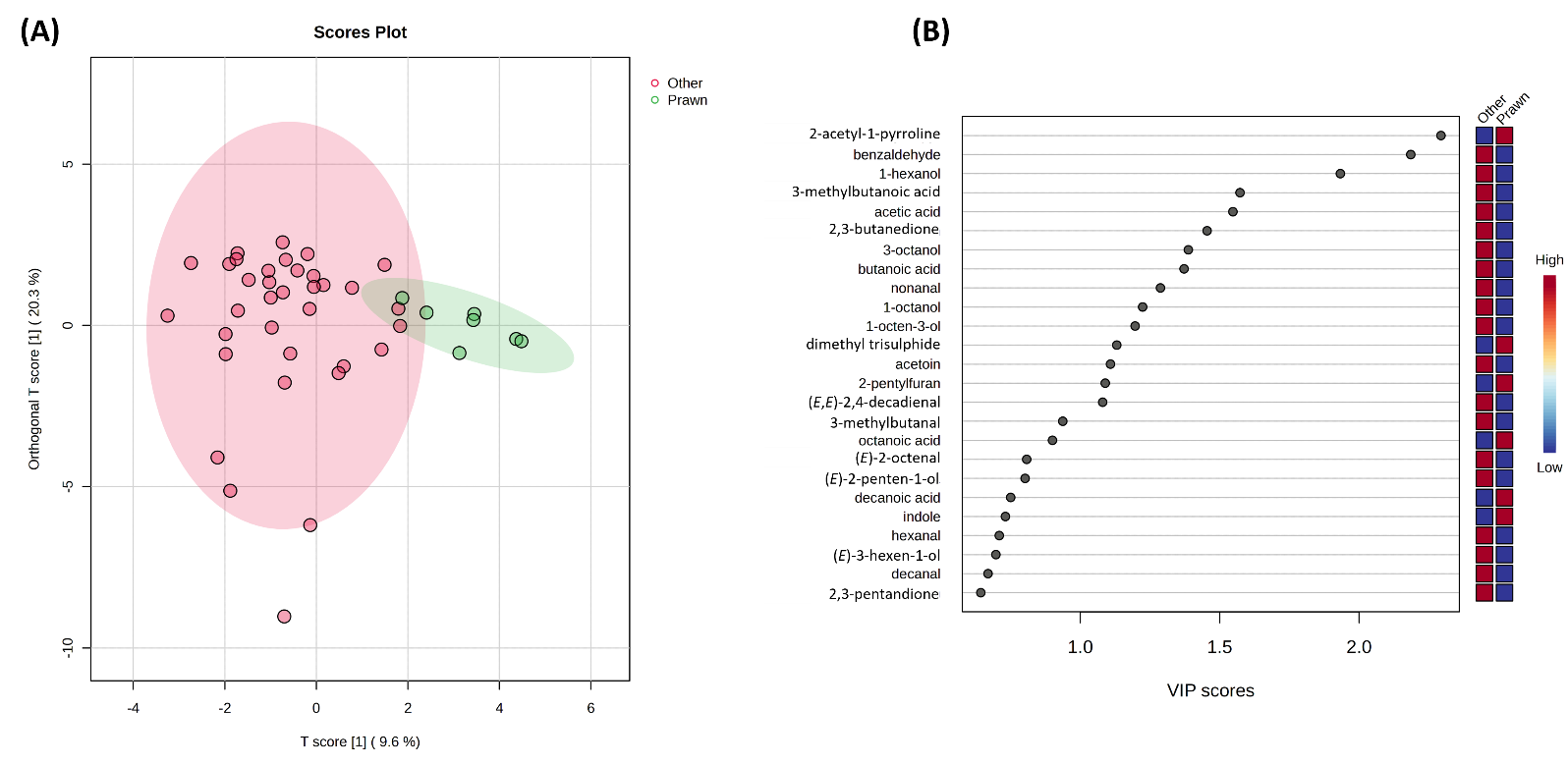


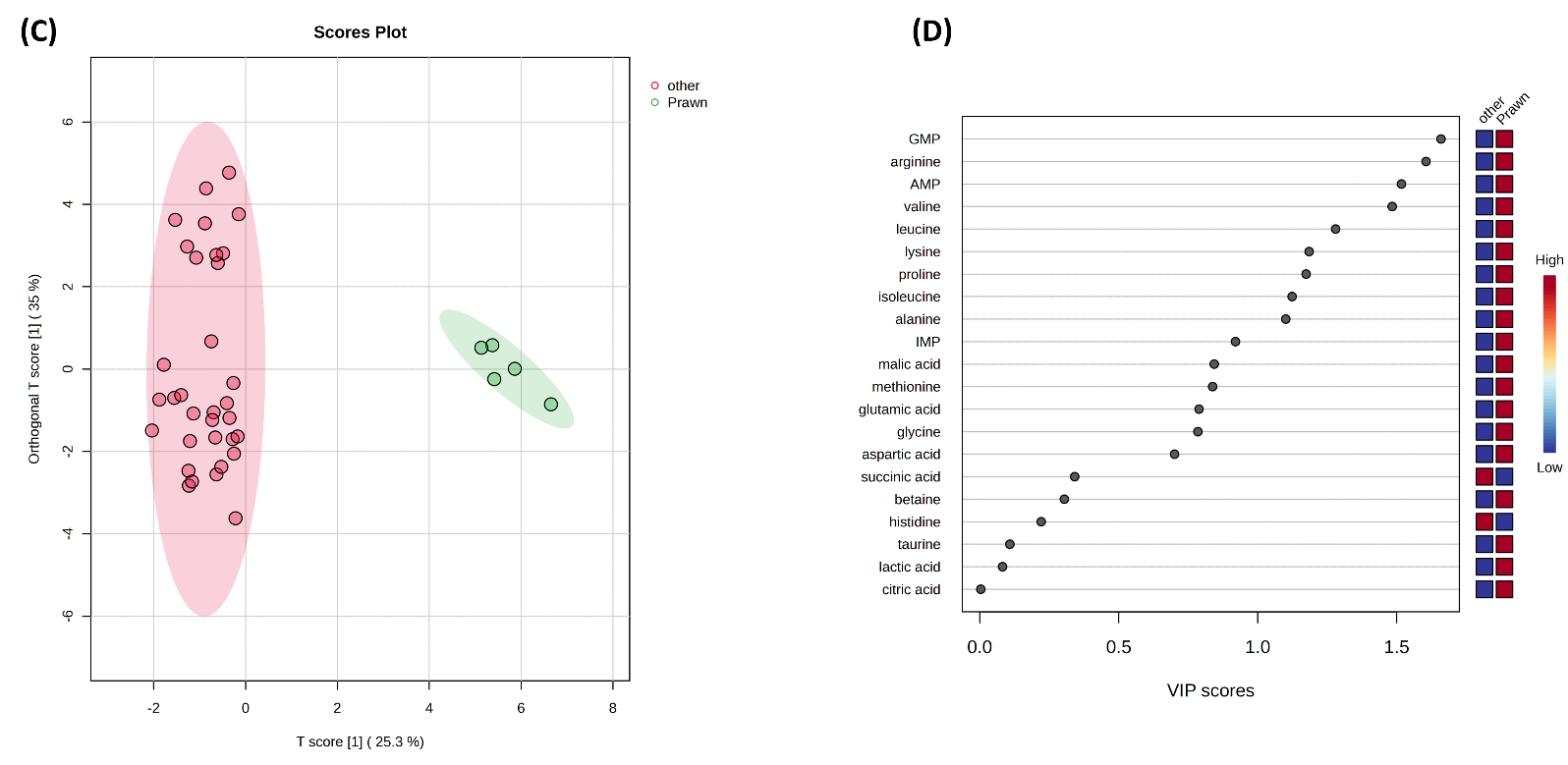


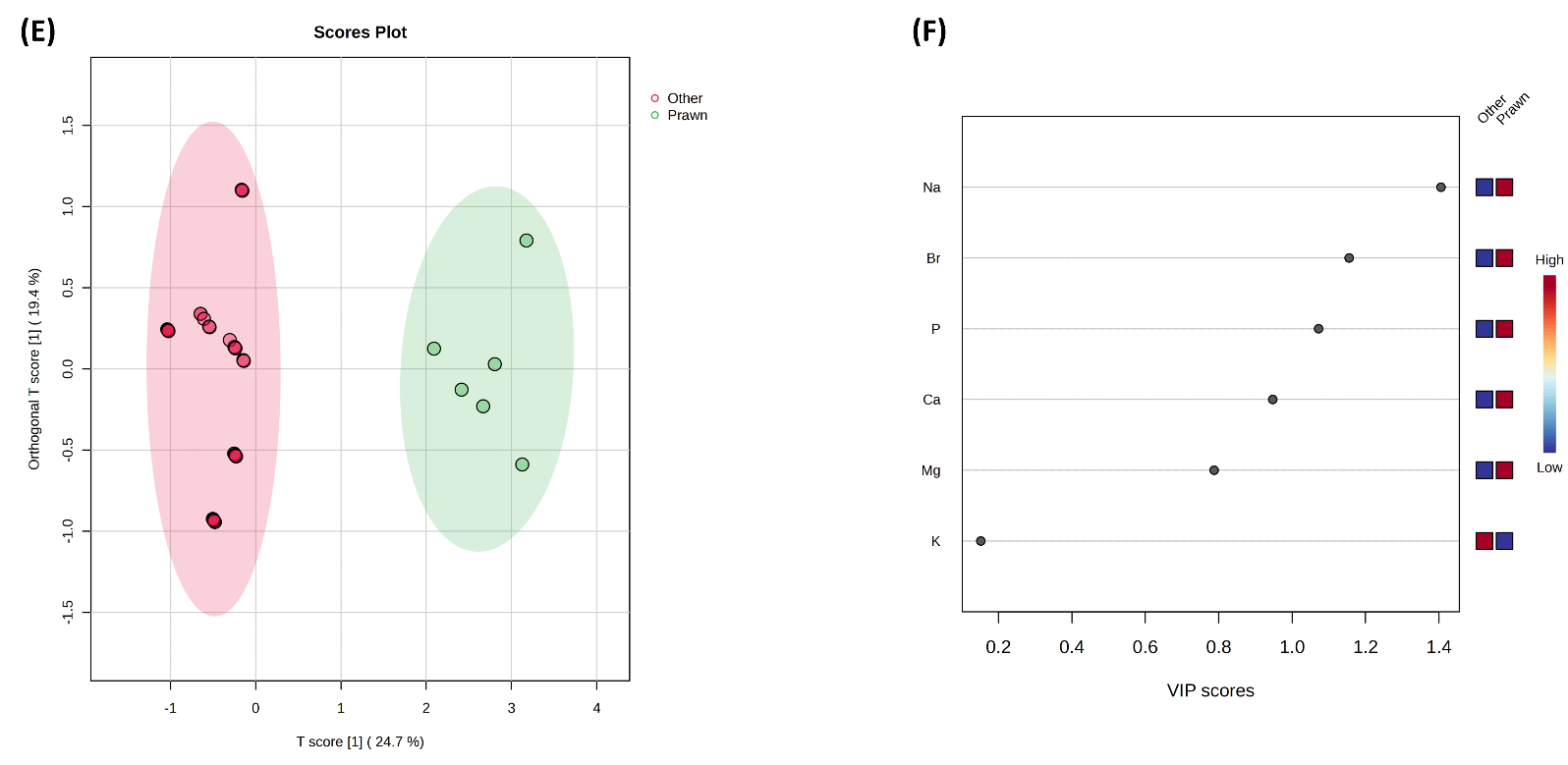


**Supplementary Figure S5.** OPLS-DA scores plots illustrate sample separation and clustering between prawn and other seafood species and rankings of VIP scores for compounds and elements. The results for odourants are displayed in panels (**A**) and (**B**), with model accuracy at Q^2^ = 0.228 and empirical p-value < 0.01 in the permutation test (*n* = 100). Tastants are shown in panels (**C**) and (**D**), with model accuracy at Q^2^ = 0.602 and empirical p-value < 0.01 in the permutation test (*n* = 100). Elements are presented in panels (**E**) and (**F**), with model accuracy at Q^2^ = 0.472 and empirical p-value < 0.01 in the permutation test (*n* = 100).


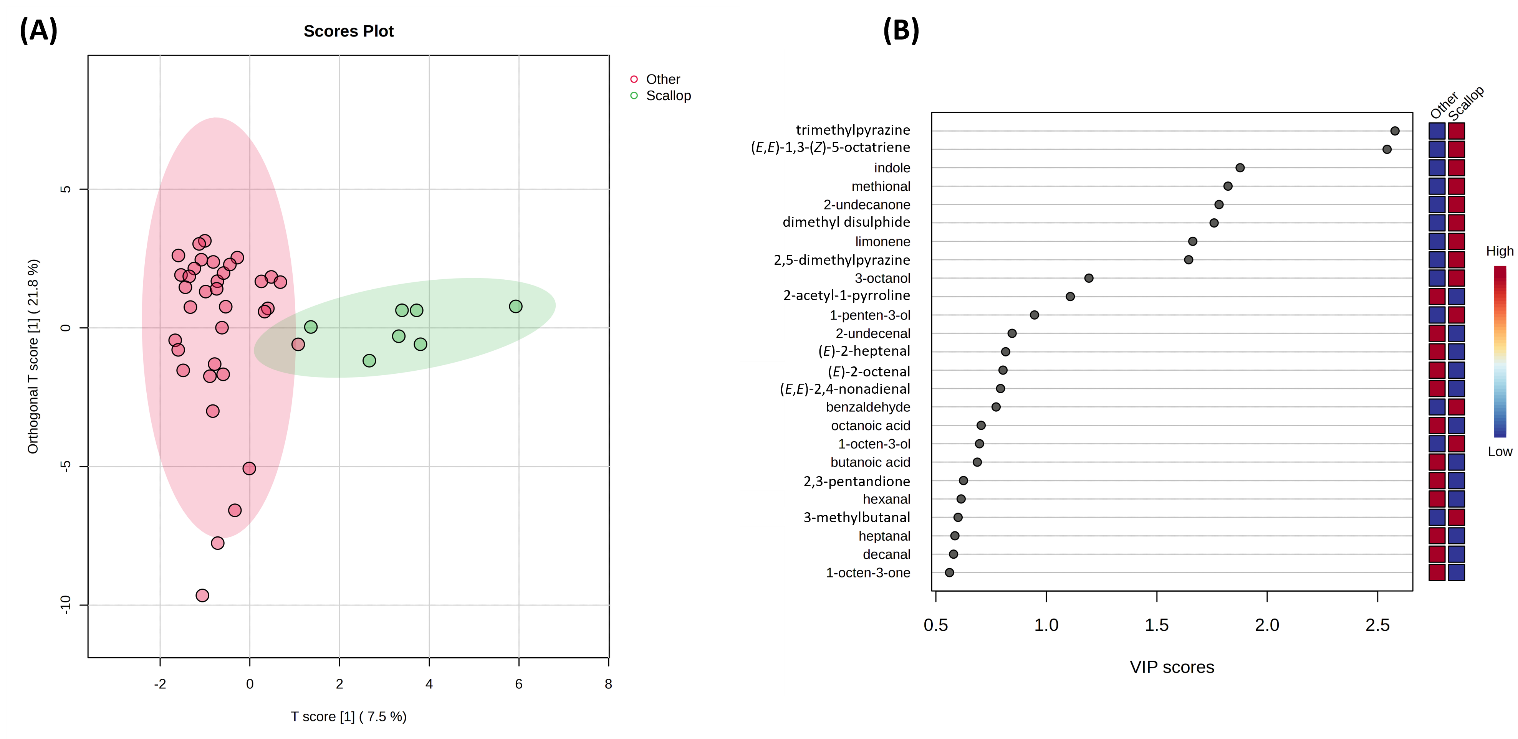


**
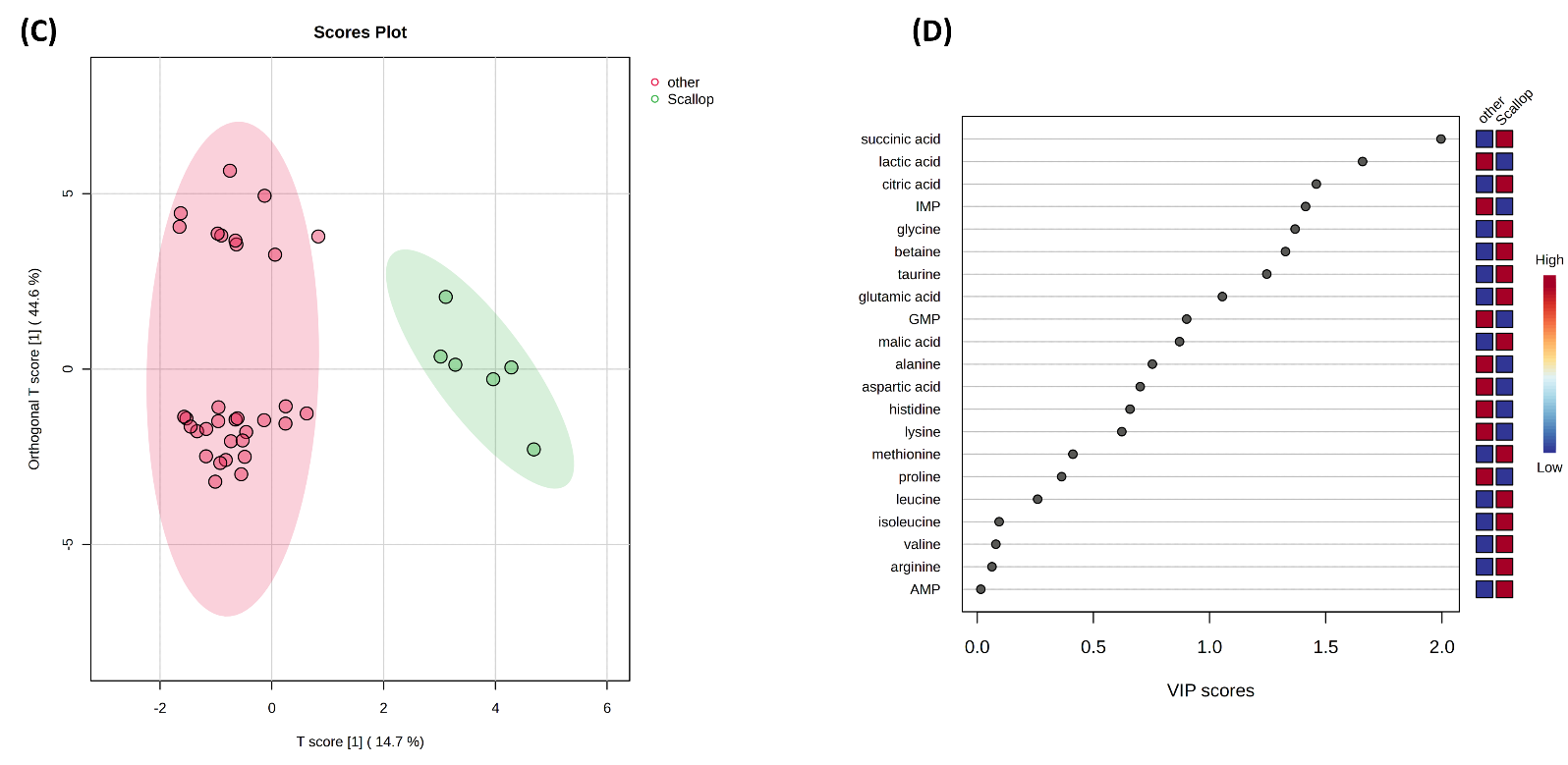
**

**
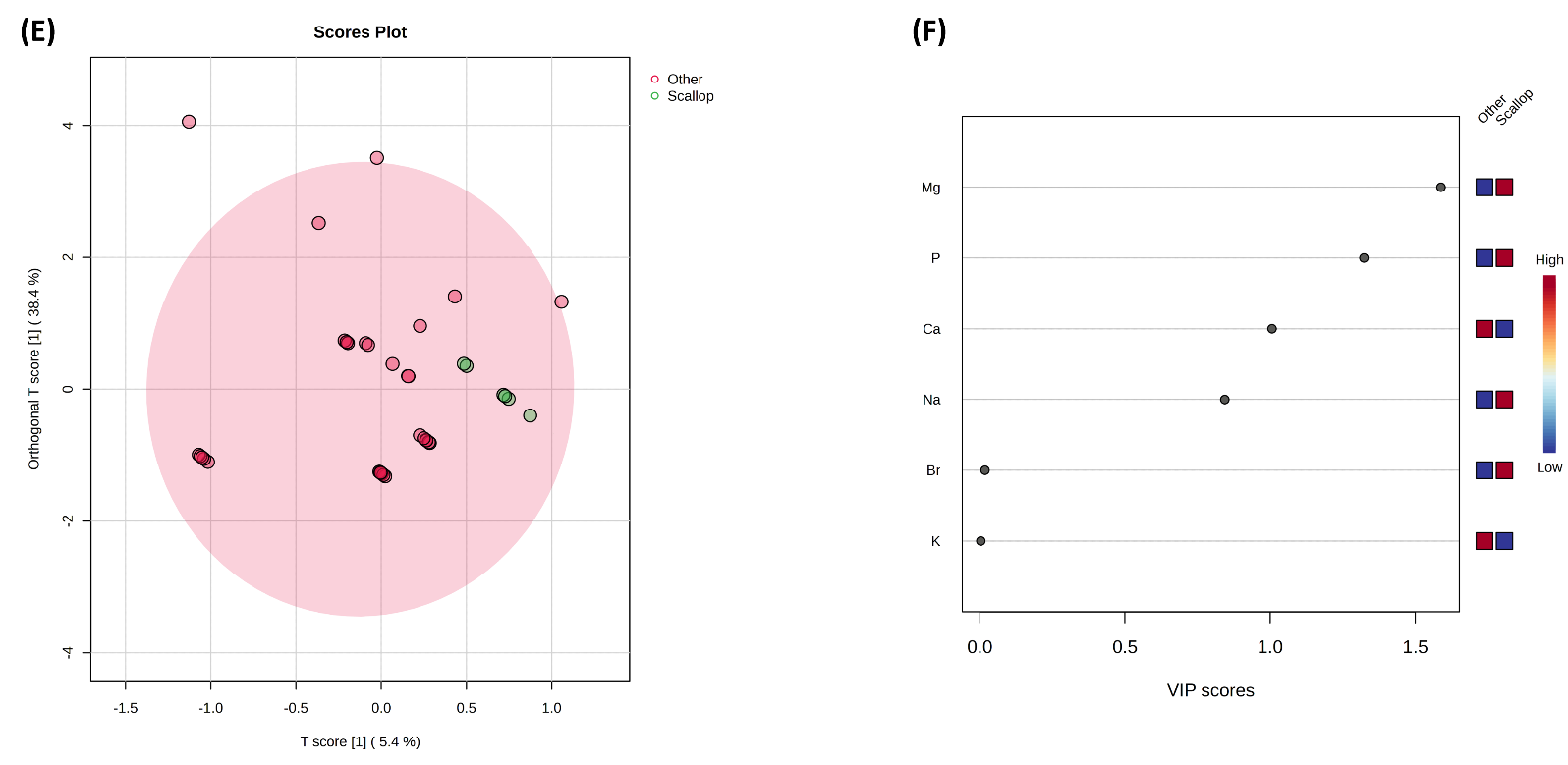
**

**Supplementary Figure S6.** OPLS-DA scores plots illustrate sample separation and clustering between scallop and other seafood species and rankings of VIP scores for compounds and elements. The results for odourants are displayed in panels (**A**) and (**B**), with model accuracy at Q^2^ = 0.355 and empirical p-value < 0.01 in the permutation test (*n* = 100). Tastants are shown in panels (**C**) and (**D**), with model accuracy at Q^2^ = 0.490 and empirical p-value < 0.01 in the permutation test (*n* = 100). Elements are presented in panels (**E**) and (**F**), with model accuracy at Q^2^ = 0.0524 and empirical p-value = 0.02 in the permutation test (*n* = 100).

**
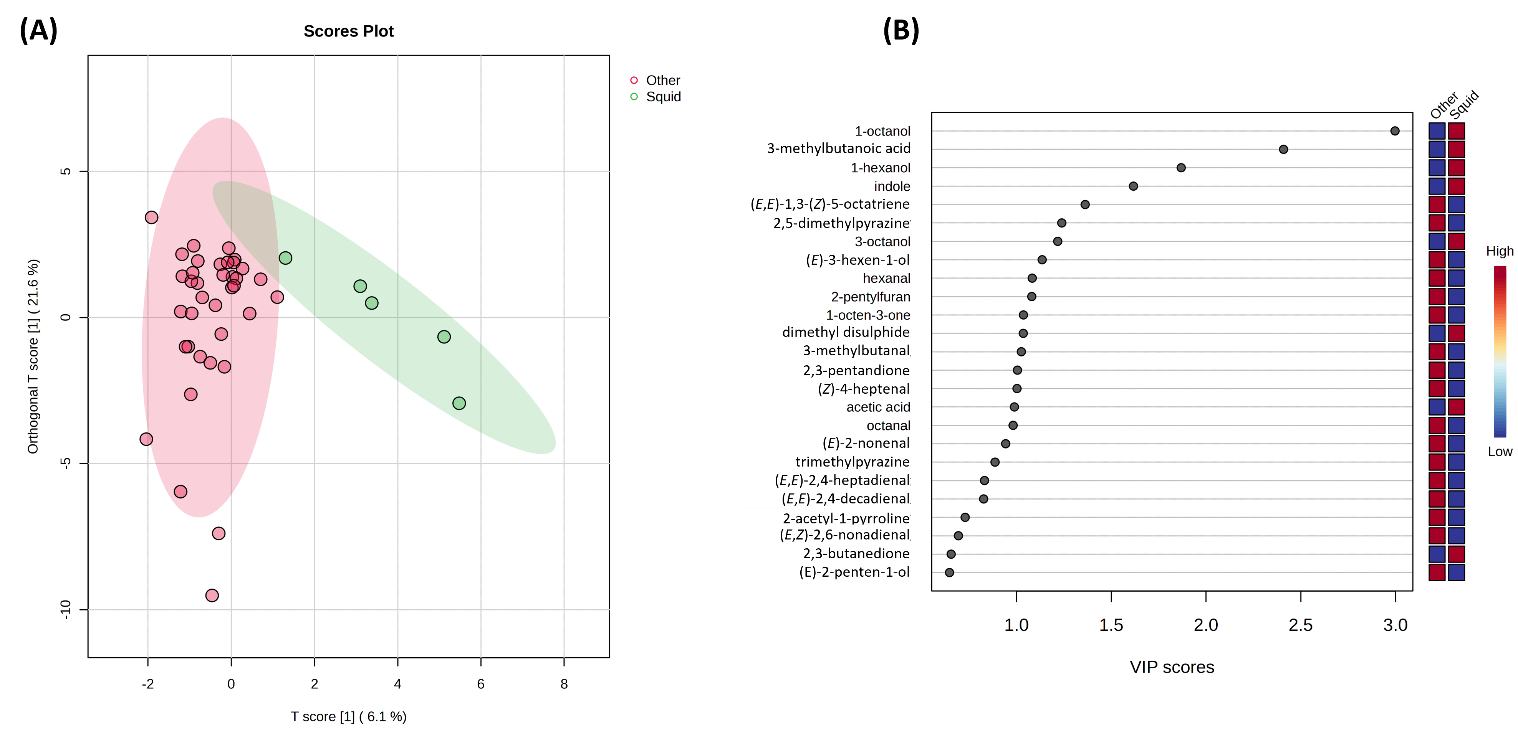
**

**
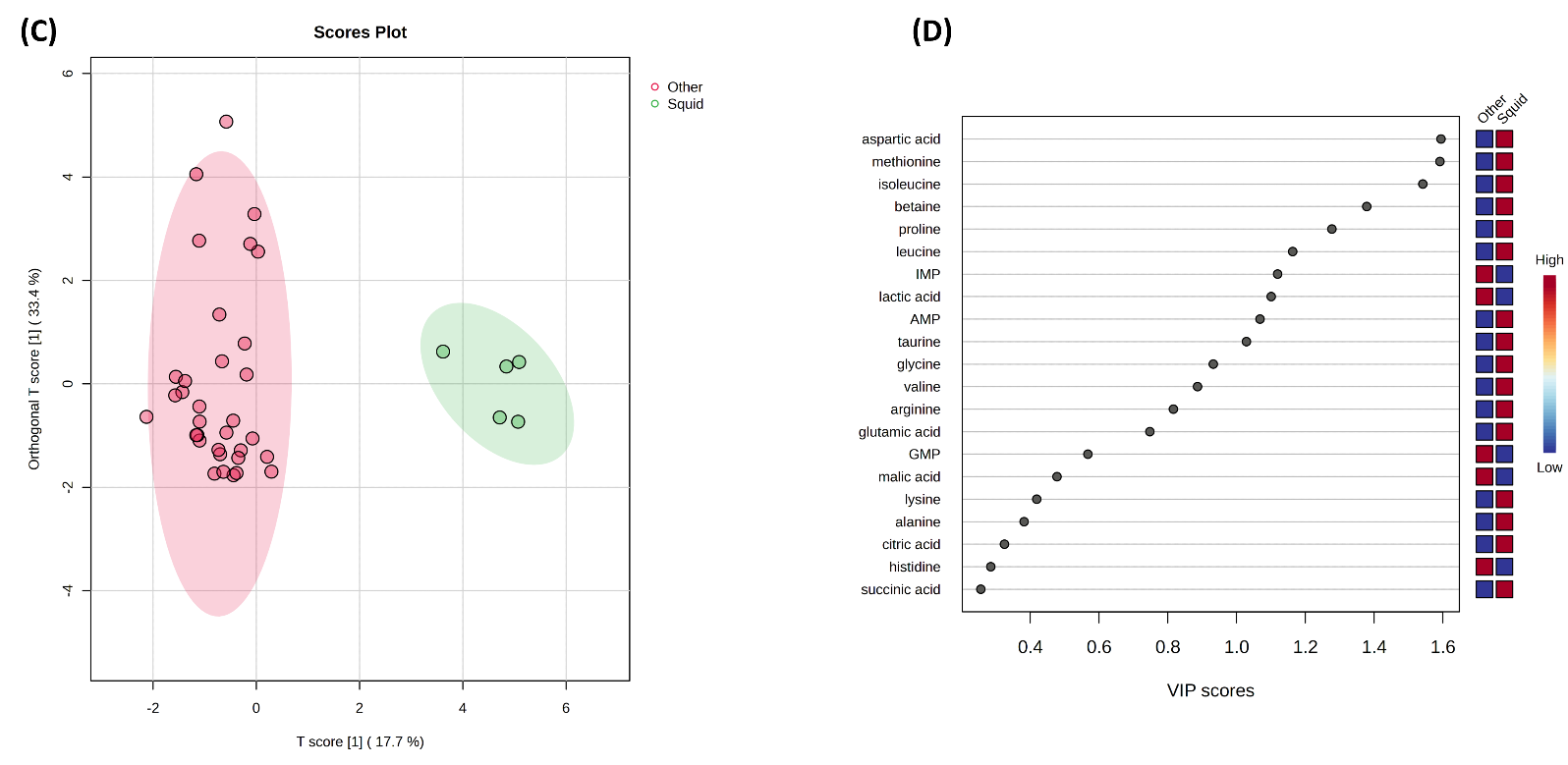
**

**
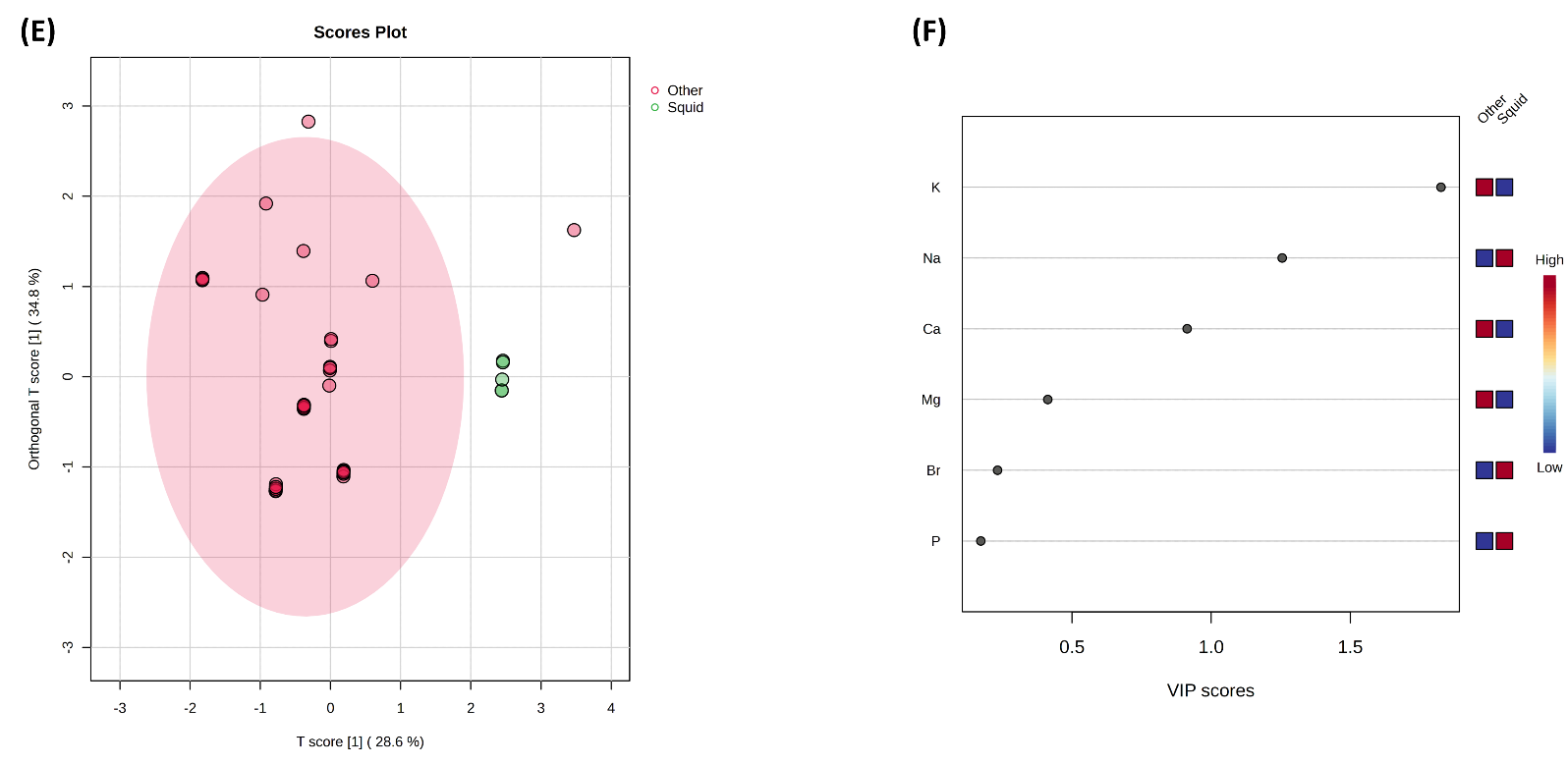
**

**Supplementary Figure S7.** OPLS-DA scores plots illustrate sample separation and clustering between squid and other seafood species and rankings of VIP scores for compounds and elements. The results for odourants are displayed in panels (**A**) and (**B**), with model accuracy at Q^2^ = 0.183 and empirical p-value < 0.01 in the permutation test (*n* = 100). Tastants are shown in panels (**C**) and (**D**), with model accuracy at Q^2^ = 0.348 and empirical p-value < 0.01 in the permutation test (*n* = 100). Elements are presented in panels (**E**) and (**F**), with model accuracy at Q^2^ = 0.379 and empirical p-value = 0.02 in the permutation test (*n* = 100).

**
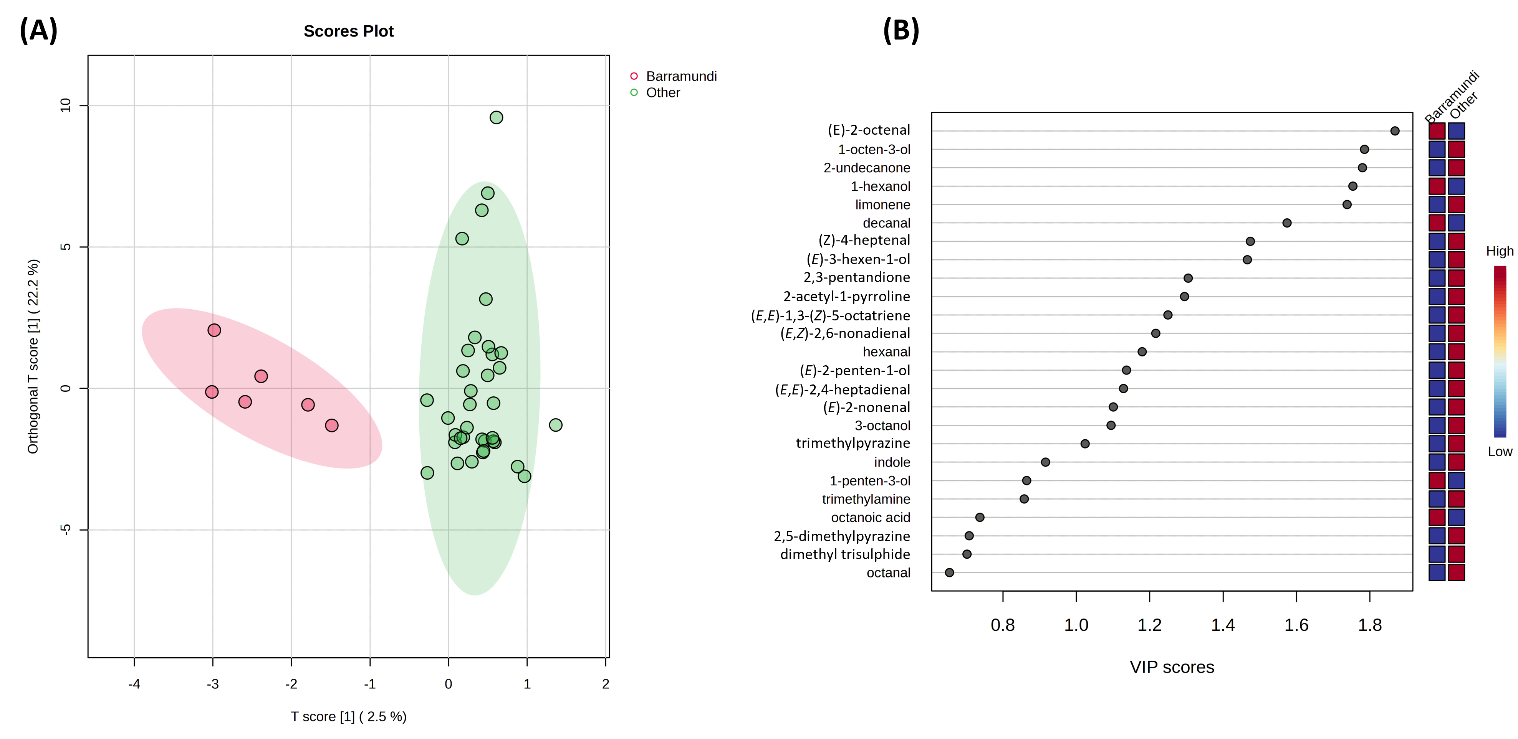
**

**
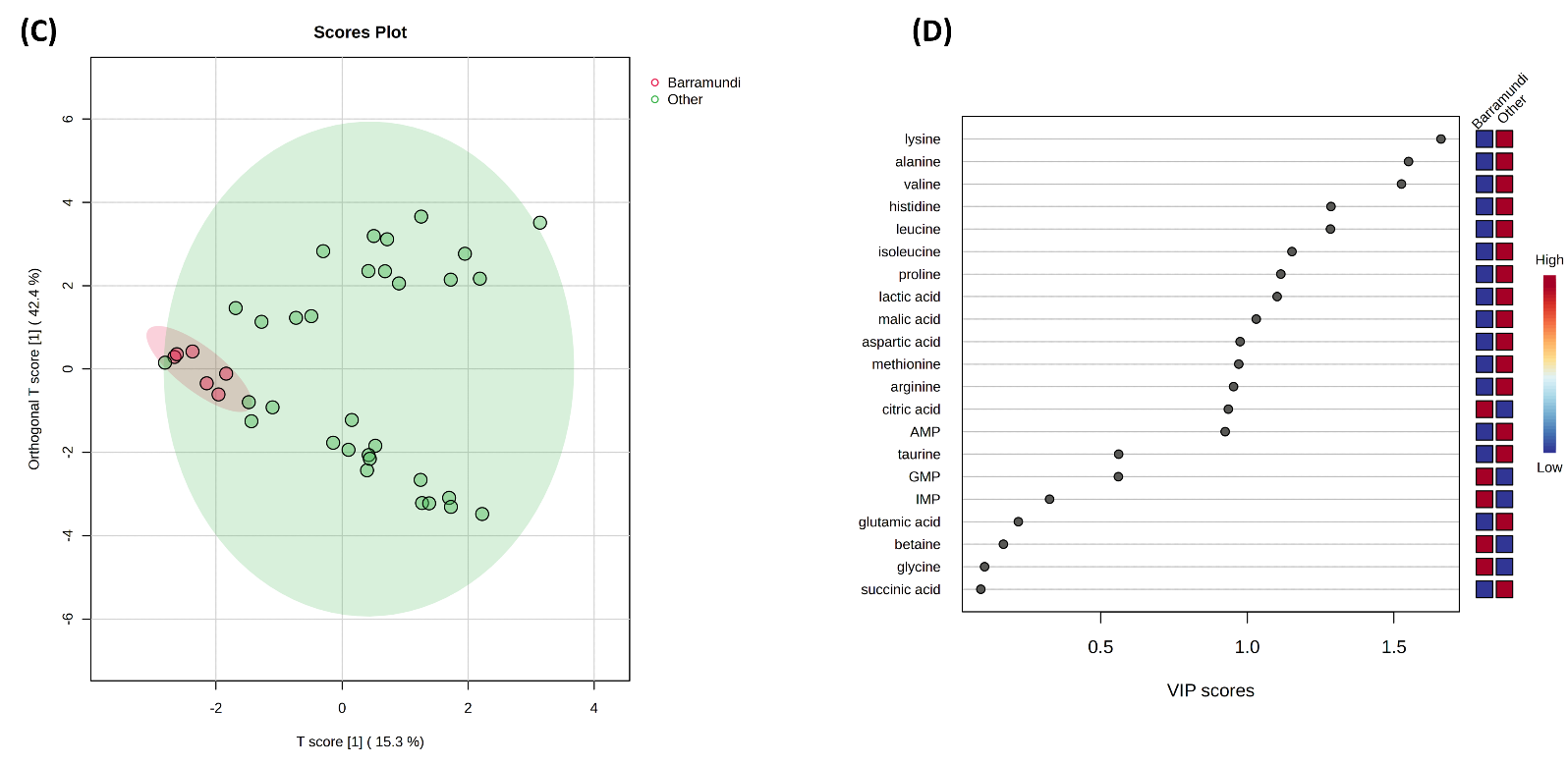
**

**
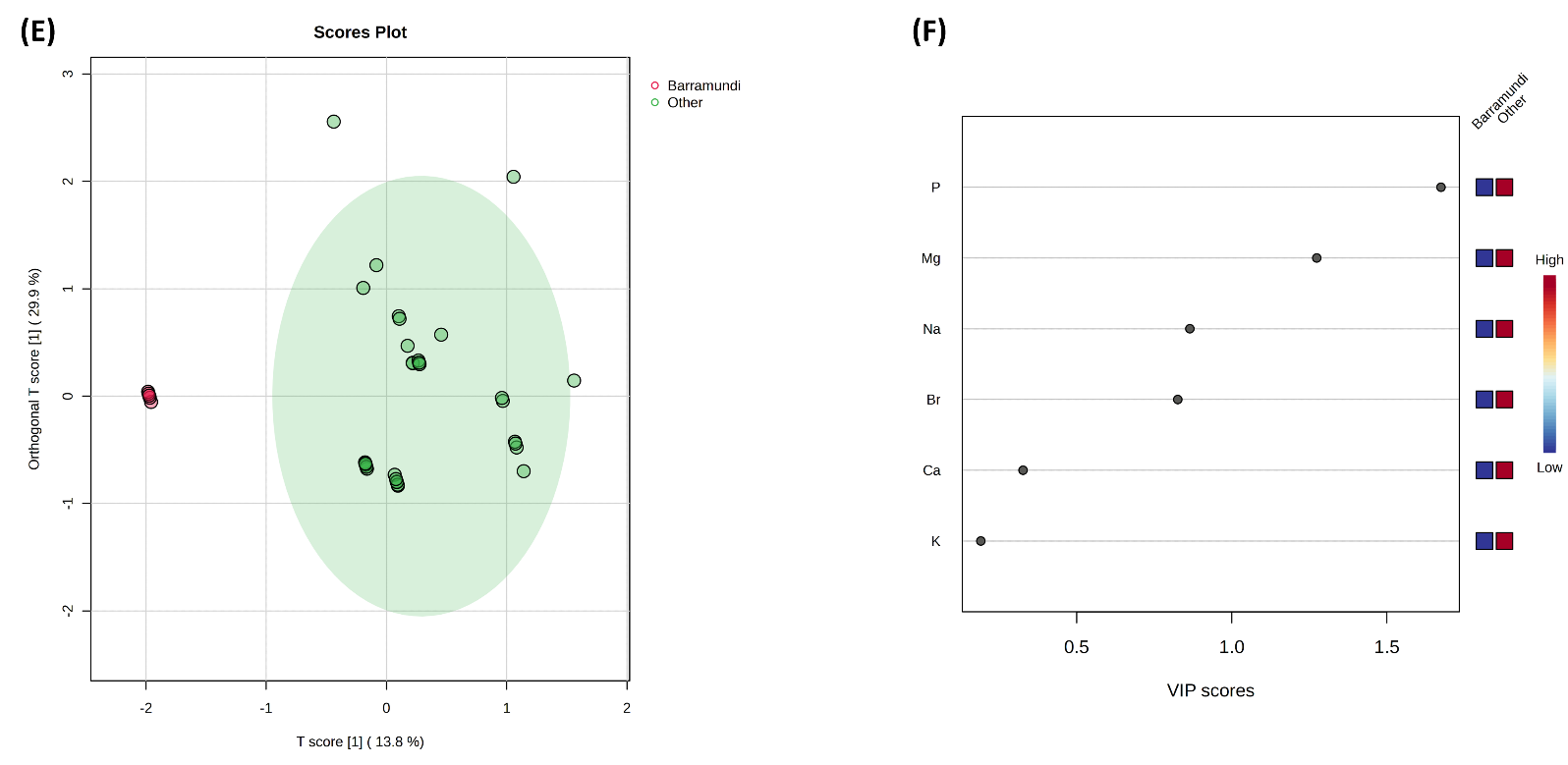
**

**Supplementary Figure S8.** OPLS-DA scores plots illustrate sample separation and clustering between barramundi and other seafood species and rankings of VIP scores for compounds and elements. The results for odourants are displayed in panels (**A**) and (**B**), with model accuracy at Q^2^ = 0.155 and empirical p-value < 0.01 in the permutation test (*n* = 100). Tastants are shown in panels (**C**) and (**D**), with model accuracy at Q^2^ = 0.135 and empirical p-value < 0.01 in the permutation test (*n* = 100). Elements are presented in panels (**E**) and (**F**), with model accuracy at Q^2^ = 0.274 and empirical p-value < 0.01 in the permutation test (*n* = 100).


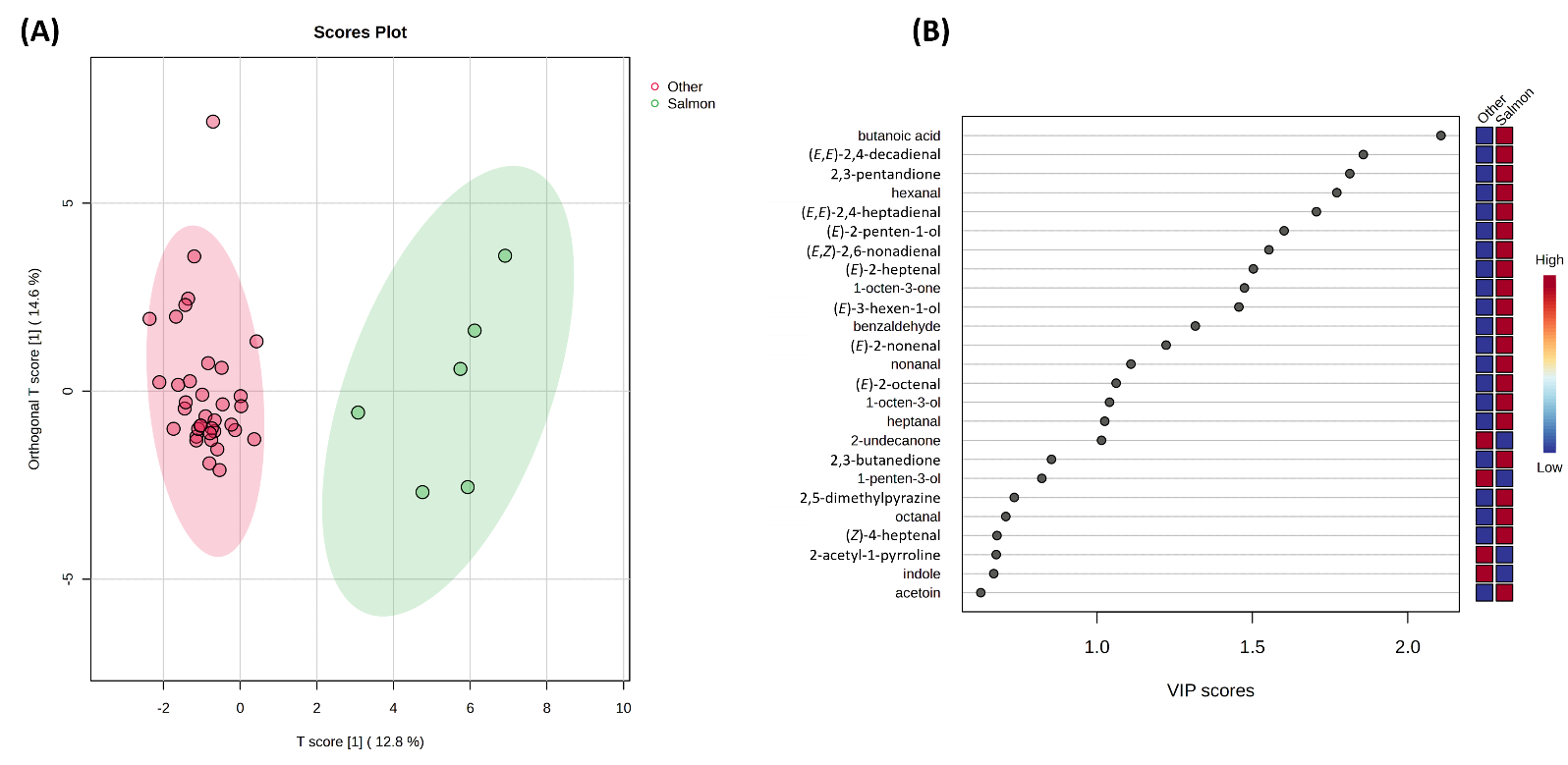


**
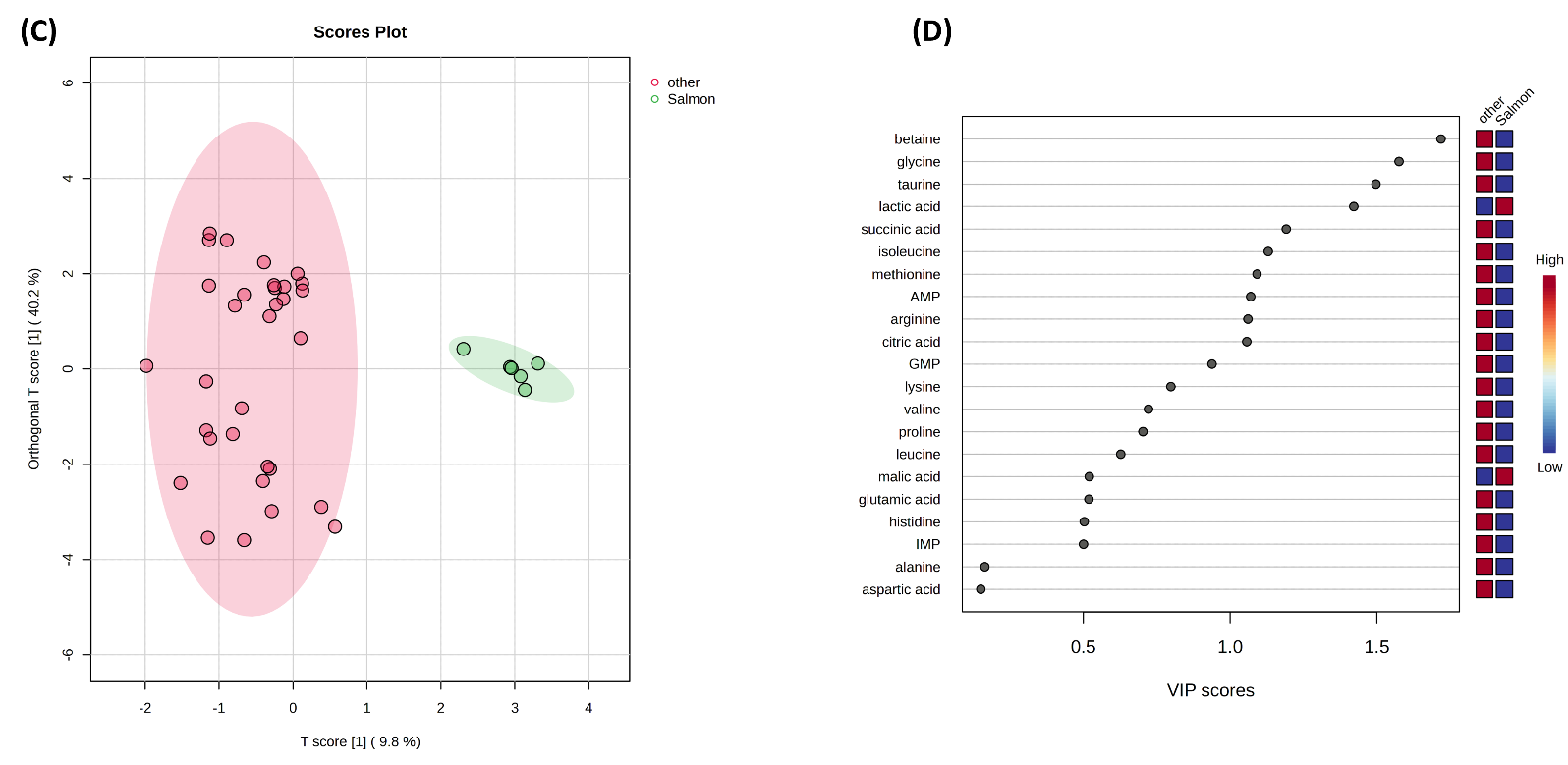
**

**
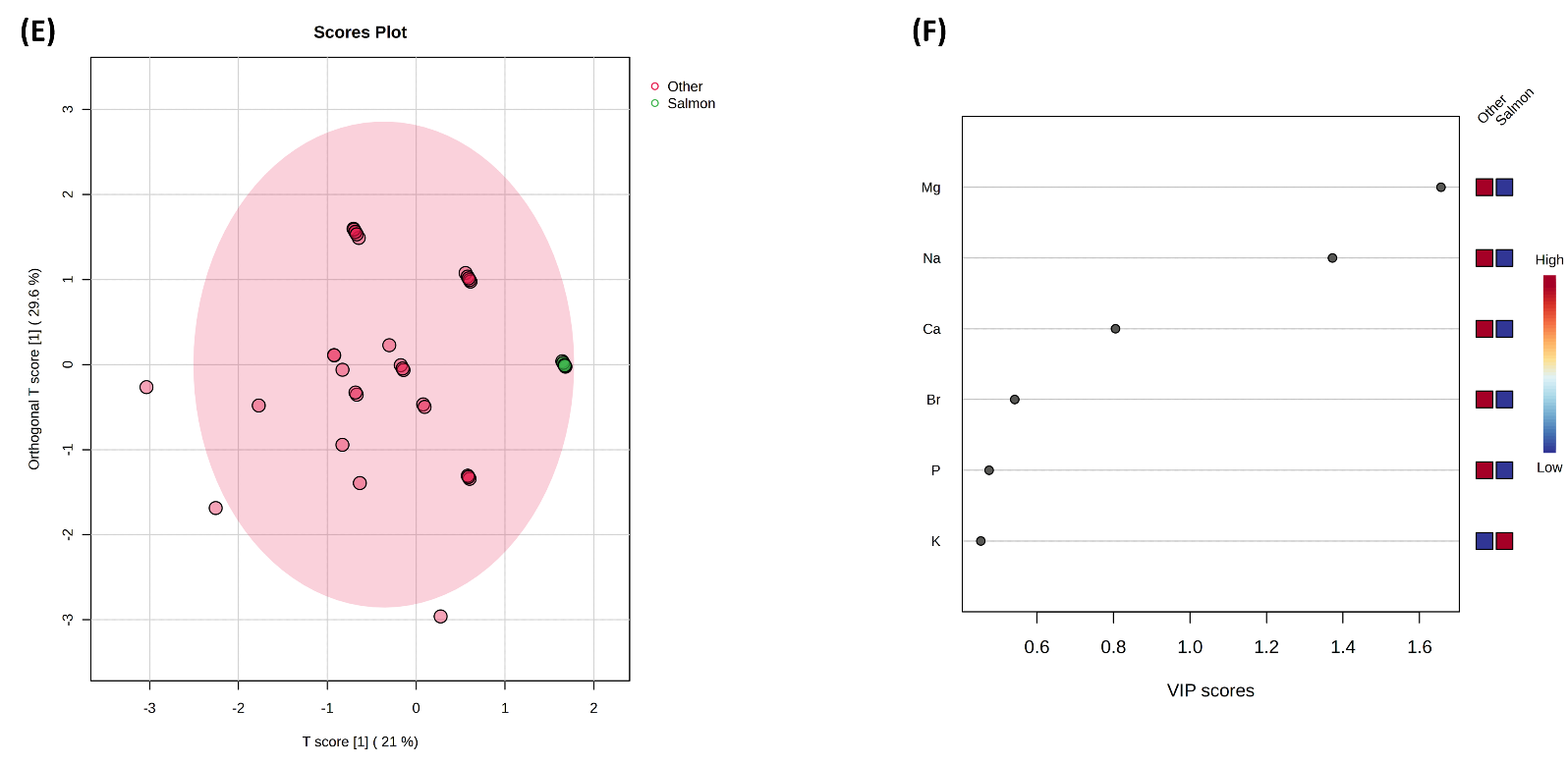
**

**Supplementary Figure S9.** OPLS-DA scores plots illustrate sample separation and clustering between salmon and other seafood species and rankings of VIP scores for compounds and elements. The results for odourants are displayed in panels (**A**) and (**B**), with model accuracy at Q^2^ = 0.440 and empirical p-value < 0.01 in the permutation test (*n* = 100). Tastants are shown in panels (**C**) and (**D**), with model accuracy at Q^2^ = 0.448 and empirical p-value < 0.01 in the permutation test (*n* = 100). Elements are presented in panels (**E**) and (**F**), with model accuracy at Q^2^ = 0.314 and empirical p-value < 0.01 in the permutation test (*n* = 100).


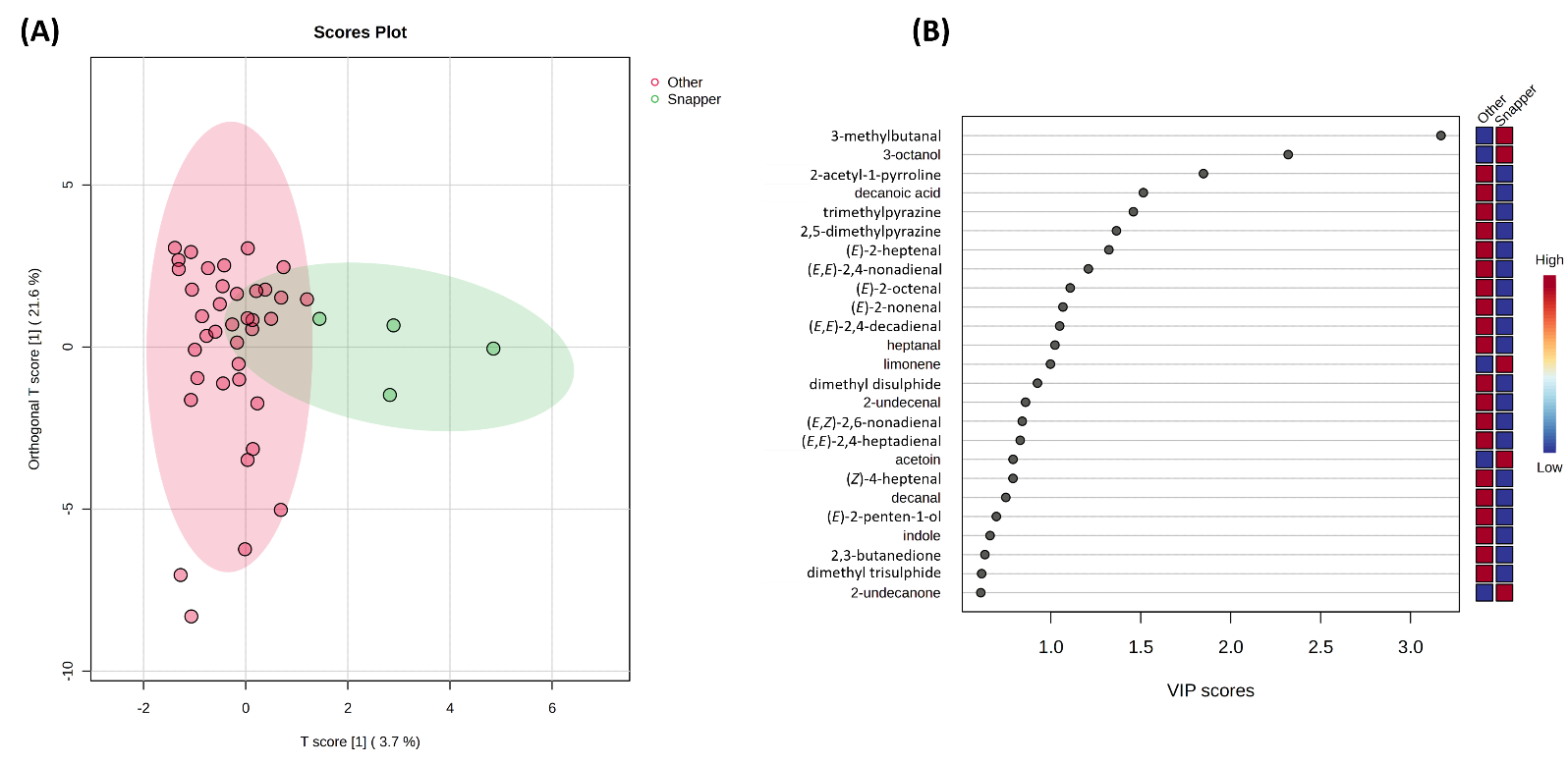


**
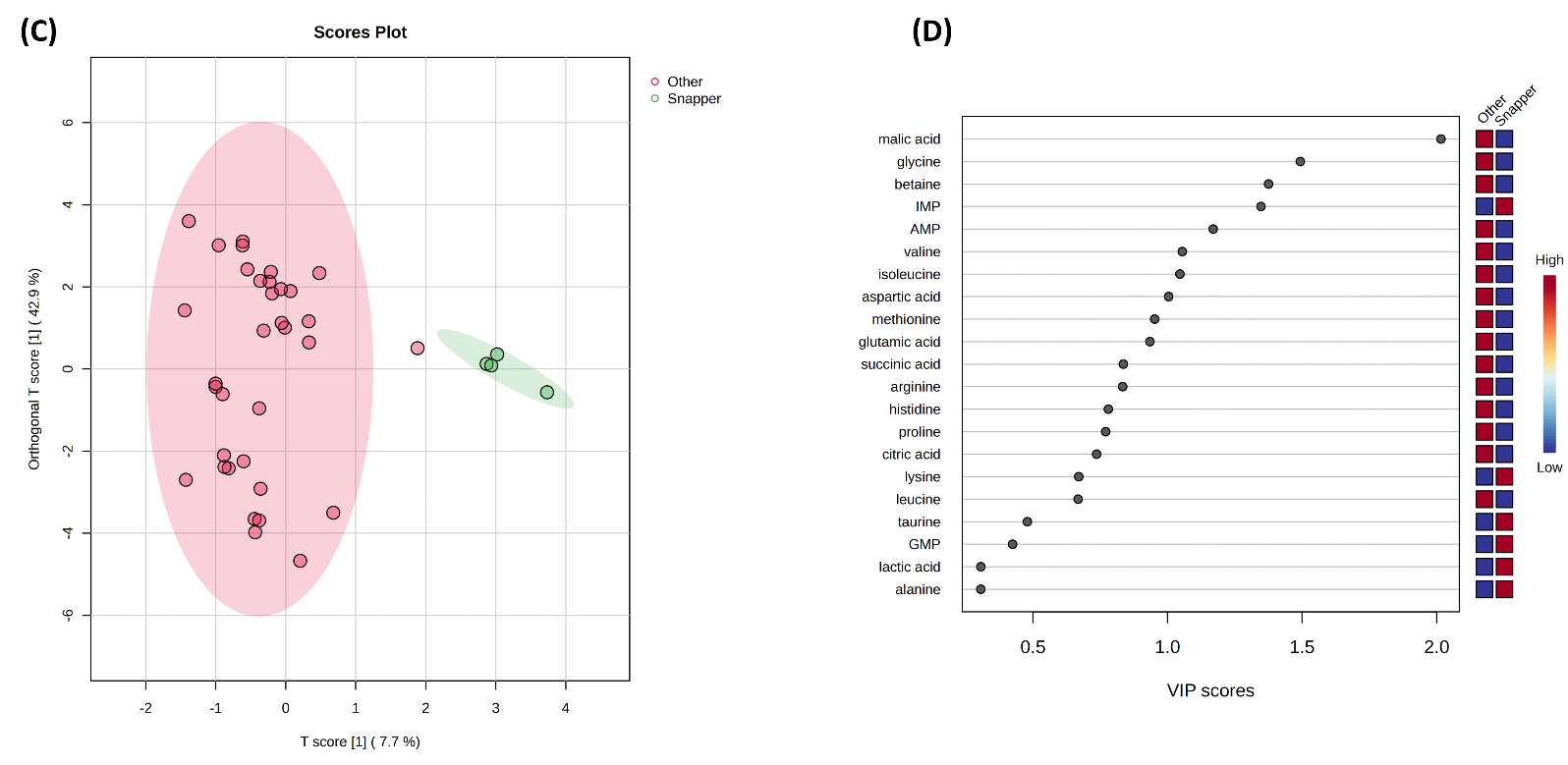
**

**
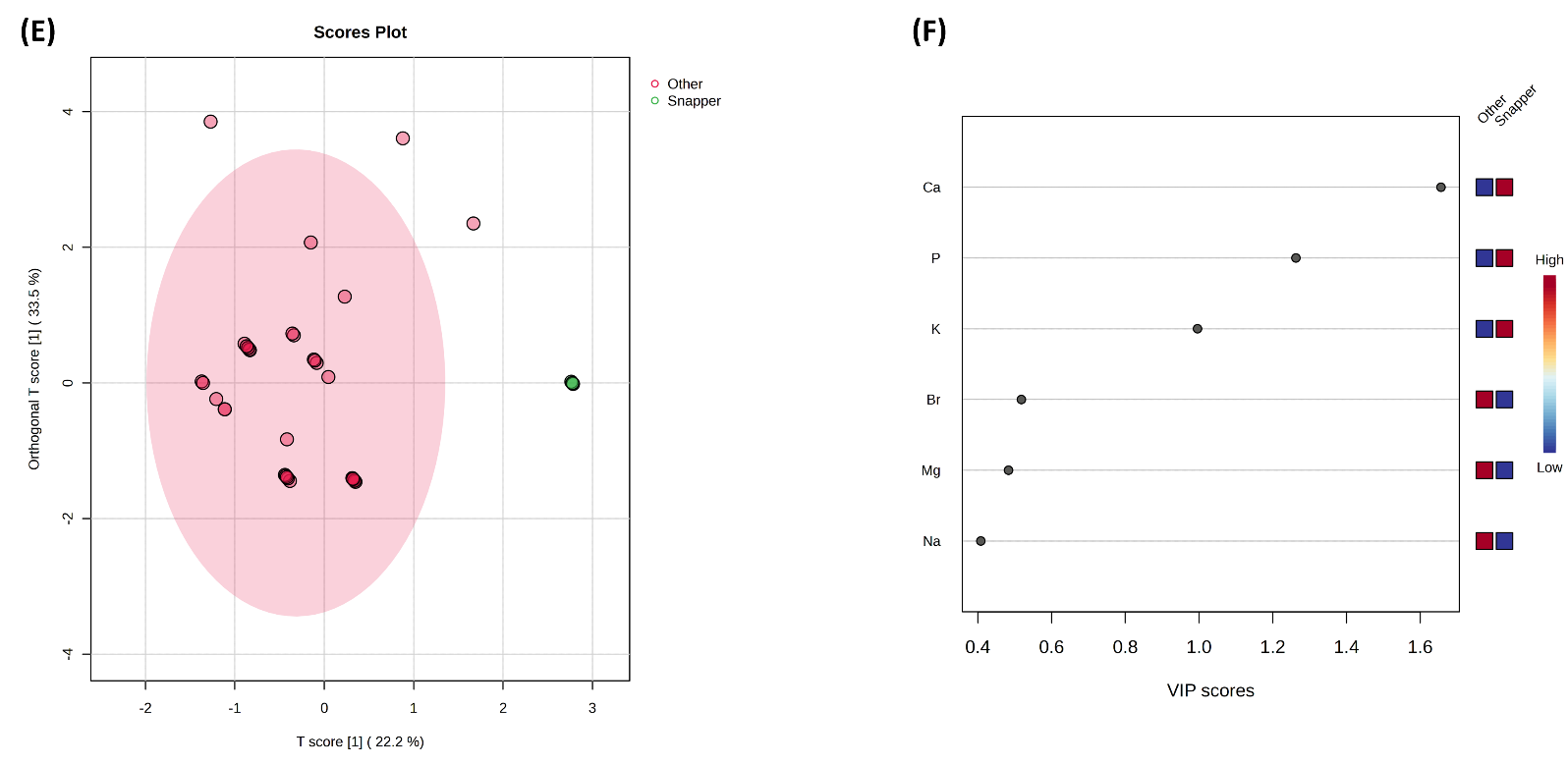
**

**Supplementary Figure S10.** OPLS-DA scores plots illustrate sample separation and clustering between snapper and other seafood species and rankings of VIP scores for compounds and elements. The results for odourants are displayed in panels (**A**) and (**B**), with model accuracy at Q^2^ = 0.0493 and empirical p-value = 0.06 in the permutation test (*n* = 100). Tastants are shown in panels (**C**) and (**D**), with model accuracy at Q^2^ = 0.311 and empirical p-value < 0.01 in the permutation test (*n* = 100). Elements are presented in panels (**E**) and (**F**), with model accuracy at Q^2^ = 0.581 and empirical p-value < 0.01 in the permutation test (*n* = 100).


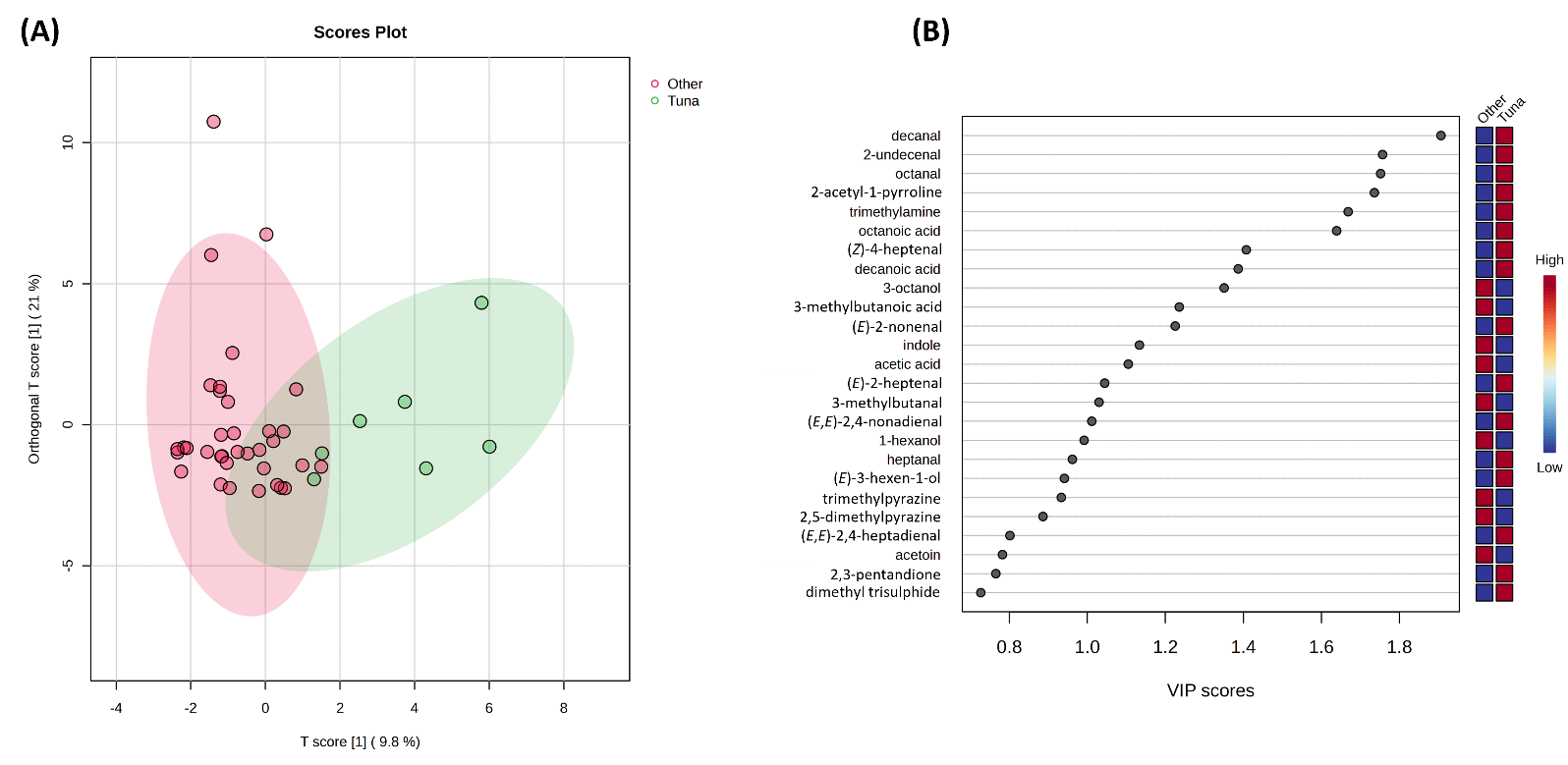


**
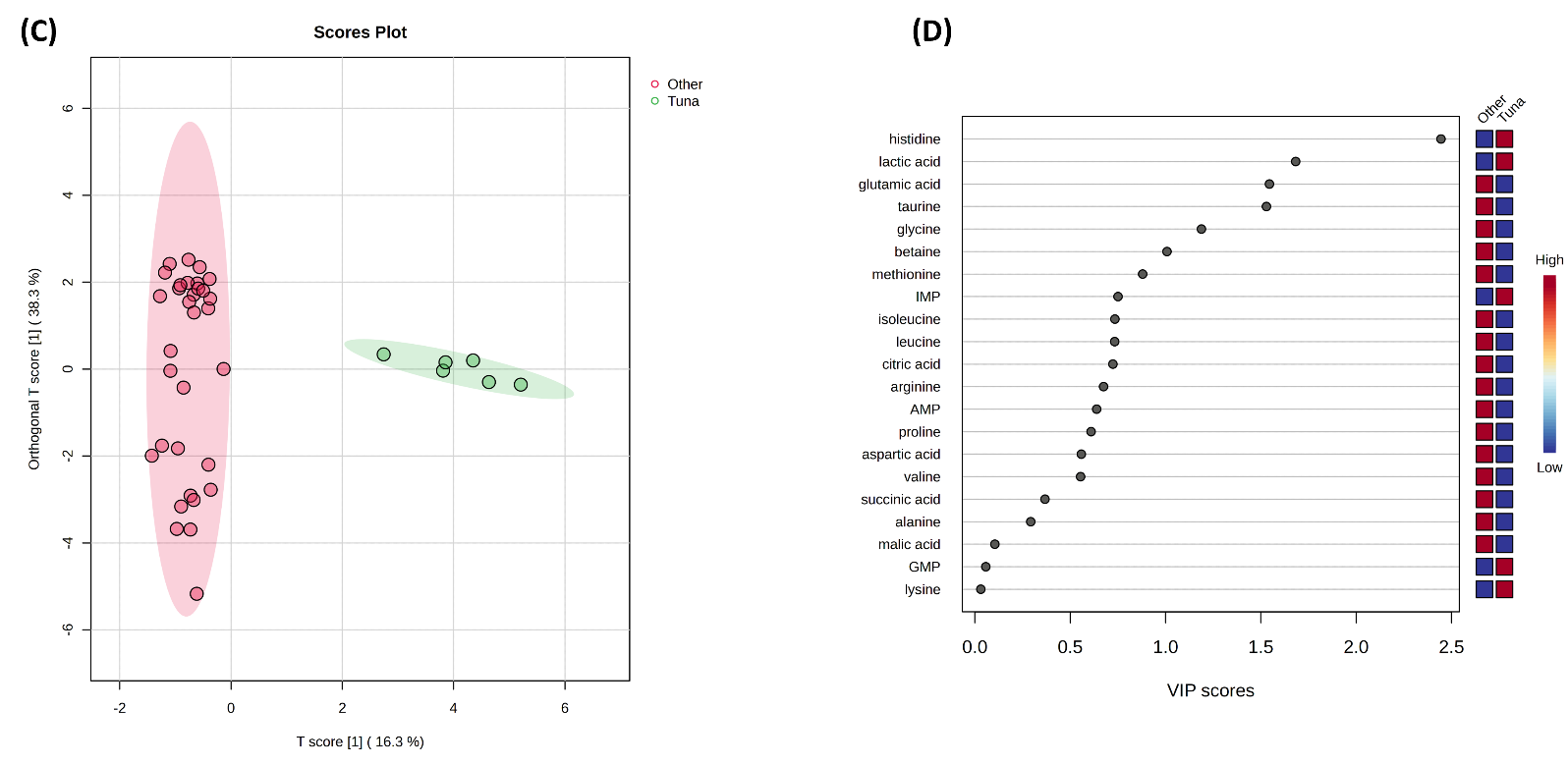
**

**
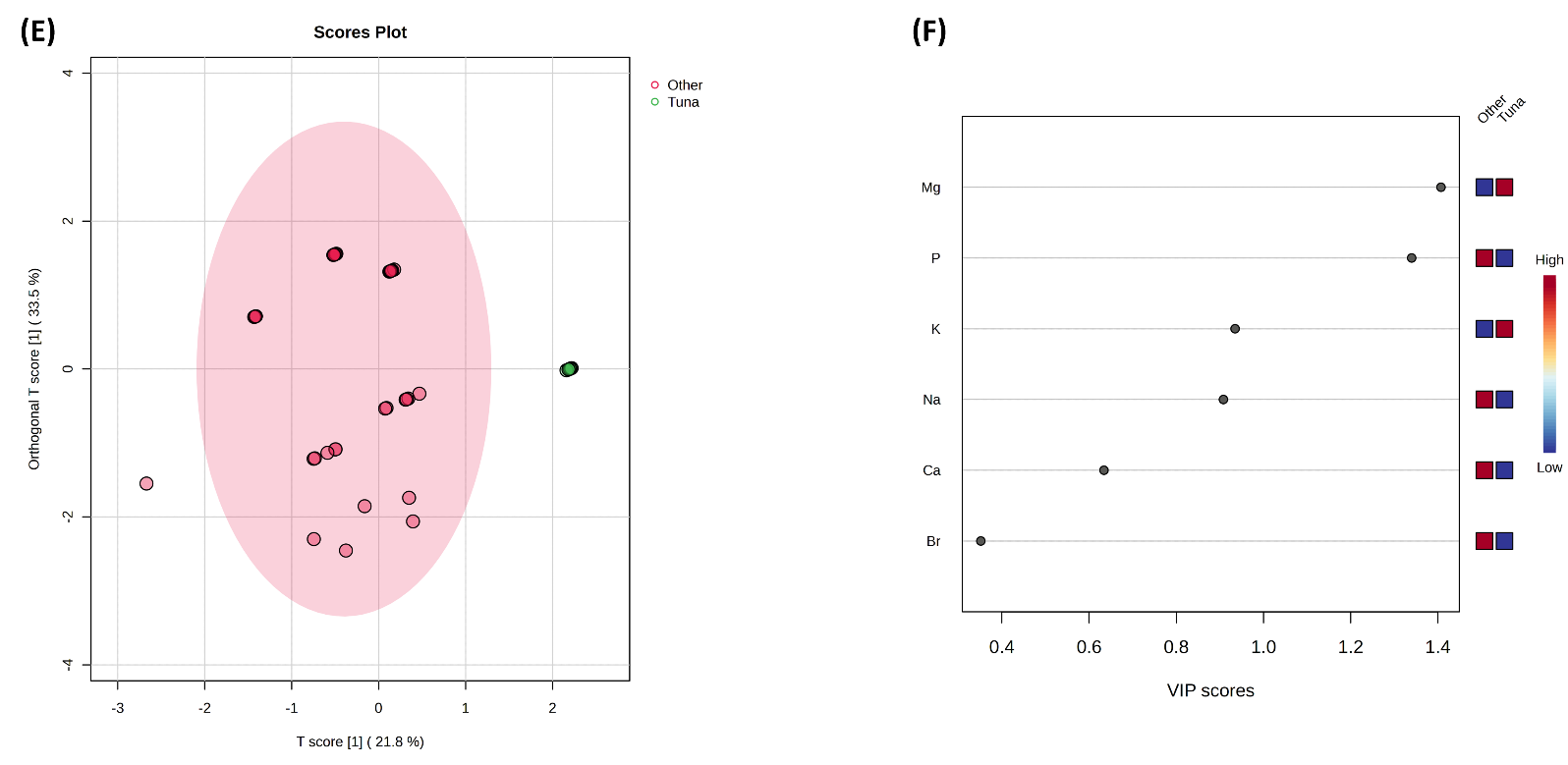
**

**Supplementary Figure S11.** OPLS-DA scores plots illustrate sample separation and clustering between tuna and other seafood species and rankings of VIP scores for compounds and elements. The results for odourants are displayed in panels (**A**) and (**B**), with model accuracy at Q^2^ = 0.283 and empirical p-value < 0.01 in the permutation test (*n* = 100). Tastants are shown in panels (**C**) and (**D**), with model accuracy at Q^2^ = 0.446 and empirical p-value < 0.01 in the permutation test (*n* = 100). Elements are presented in panels (**E**) and (**F**), with model accuracy at Q^2^ = 0.524 and empirical p-value < 0.01 in the permutation test (*n* = 100).

**References**

Chung, S., & Chan, B. (2009). Trimethylamine oxide, dimethylamine, trimethylamine and formaldehyde levels in main traded fish species in Hong Kong. *Food Additives and Contaminants: Part B*, *2*(1), 44-51.

Jääskeläinen, E., Jakobsen, L. M., Hultman, J., Eggers, N., Bertram, H. C., & Björkroth, J. (2019). Metabolomics and bacterial diversity of packaged yellowfin tuna (Thunnus albacares) and salmon (Salmo salar) show fish species-specific spoilage development during chilled storage. *International Journal of Food Microbiology*, *293*, 44-52.

Kelly, R. H., & Yancey, P. H. (1999). High contents of trimethylamine oxide correlating with depth in deep-sea teleost fishes, skates, and decapod crustaceans. *The Biological Bulletin*, *196*(1), 18-25.

Ruiz-Capillas, C., Horner, W., & Gillyon, C. (2001). Effect of packaging on the spoilage of king scallop (Pecten maximus) during chilled storage. *European Food Research and Technology*, *213*, 95-98.
